# Formation, Characterization, and Occurrence of β-Carboline Alkaloids Derived from α-Dicarbonyl Compounds and l-Tryptophan

**DOI:** 10.1021/acs.jafc.2c03187

**Published:** 2022-07-12

**Authors:** Tomás Herraiz, Adriana Peña, Haroll Mateo, Marta Herraiz, Antonio Salgado

**Affiliations:** † Spanish National Research Council (CSIC), Instituto de Ciencia y Tenología de Alimentos y Nutrición (ICTAN-CSIC), Jose Antonio Novais 10, Ciudad Universitaria, 28040 Madrid, Spain; ‡ Centro de Espectroscopía de RMN (CERMN), Universidad de Alcalá (UAH), Campus Universitario Ctra. Madrid-Barcelona km 33.6, 28805 Alcalá de Henares, Madrid, Spain

**Keywords:** α-dicarbonyls, β-carboline alkaloids, α-dicarbonyl-derived βCs, tryptophan, glyoxal, methylglyoxal, 3-deoxyglucosone, Maillard reaction, advanced glycation

## Abstract

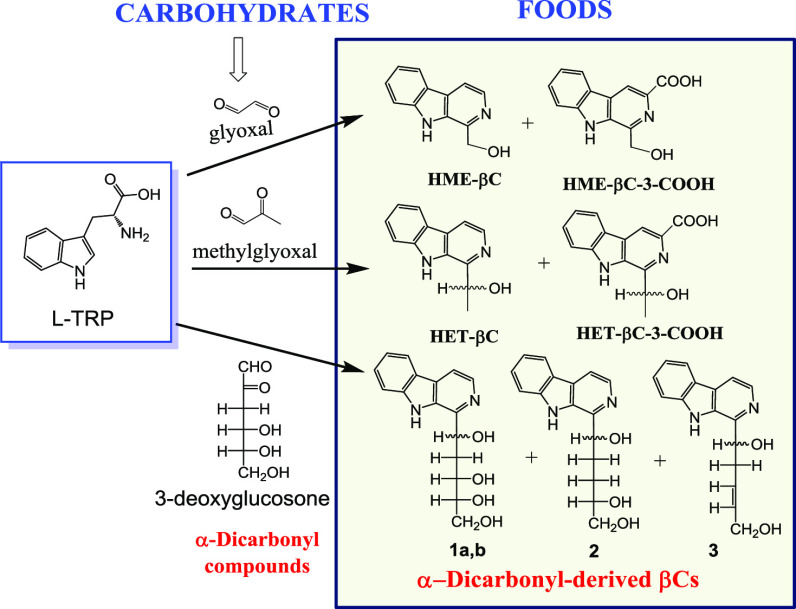

β-Carbolines (βCs) are naturally occurring bioactive alkaloids, whereas α-dicarbonyl compounds are reactive substances generated in foods and *in vivo*. In this work, l-tryptophan reacted with α-dicarbonyl compounds affording new β-carbolines. Glyoxal afforded 1-hydroxymethyl-β-carboline (HME-βC) and its 3-carboxylic acid, and methylglyoxal afforded 1-(1-hydroxyethyl)-β-carboline (HET-βC) and its 3-carboxylic acid. 3-Deoxyglucosone afforded 1-(1,3,4,5-tetrahydroxypent-1-yl)-β-carboline isomers (**1a/b**), 1-(1,4,5-trihydroxypent-1-yl)-β-carboline (**2**), and 1-(1,5-dihydroxypent-3-en-1-yl)-β-carboline (**3**). The formation of these βCs increased under acidic conditions and with increasing temperature. A mechanism is proposed explaining the conversion of a carbonyl into a hydroxy group based on tautomerism and cyclization to the dihydro-βC-3-COOH intermediates, which were isolated and gave the βCs. These α-dicarbonyl-derived βCs occurred in model reactions of l-tryptophan with fructose or glucose incubated under heating and can be considered as advanced glycation end products (AGEs). They were also present in foods and formed during heating processes. HET-βC appeared in processed foods, reaching up to 309 ng/g, with the highest amount found in dried tomato, fried onion, toasted bread, and Manuka honey. HME-βC was only detected in some foods with lower amounts than HET-βC. HET-βC appeared in foods as a racemic mixture of enantiomers suggesting the same mechanism of formation as the synthetized product. α-Dicarbonyl-derived βCs (HET-βC, HME-βC, and **1a/b-3**) occur in foods and food processing and, therefore, they are ingested during diet.

## Introduction

β-Carbolines (9*H*-pyrido[3,4-*b*]indole) (βCs) are indole alkaloids that occur in foods, plants, and biological fluids and tissues.^1,2^ These alkaloids exhibit an array of biological, pharmacological, and toxicological activities. They act on the central nervous system (CNS) through serotonin uptake, benzodiazepine receptor, and imidazoline binding sites and also interact with key enzymes (*e.g.*, monoamine oxidase (MAO) and kinases).^3^ Some βCs such as norharman and harman exhibit antidepressant and behavioral effects associated with changes in neurotransmitter levels and inhibition of MAO.^4−6^ The βCs occurring in foods and cigarette smoke are potent inhibitors of MAO.^7,8^ Some βCs have been described as neuroprotective/neurogenesis agents,^9^ while others could be bioactivated by N-methylation affording endogenous neurotoxins (i.e., β-carbolinium cations) that resemble the neurotoxin MPTP.^3^ In addition, βCs are comutagenic, bind to DNA, and react with hydroxyl radicals (OH^·^)^10,11^ exhibiting radical scavenging activity. Therefore, the βCs exhibit significant bioactive and toxic actions and they can occur in tissues and biological fluids so that the exposure to these compounds via foods is a matter of interest.

The βCs are classified into tetrahydro-β-carbolines (THβCs) and aromatic β-carbolines (βCs).^1,2^ THβCs are formed through Pictet–Spengler reaction from indole-ethylamines or tryptophan and carbonyl compounds (aldehydes or α-keto acids).^2^ They have been reported in many foods, and the most abundant are the tetrahydro-β-carboline-3-carboxylic acids (THβC-3-COOH) coming from tryptophan.^1,12^ The Pictet–Spengler reaction also occurs when tryptophan reacts with glucose to give pentahydroxypentyl (PHP)-THβC-3-COOH.^13−15^ PHP-THβC-3-COOHs have been reported in foods with concentrations of up to 6.5 μg/g determined in tomato products, fruit juices, and jams^14^ and also found in human urine.^16,17^ Aromatic βCs occurring in foods or *in vivo* arise from the oxidation of THβCs.^18^ Among them, the two more relevant are norharman and harman that have been reported in foods and cigarette smoke, which are also generated in meats and fish along with heterocyclic aromatic amines during cooking.^18,19^ Moreover, several aromatic βCs derived from glucose have been reported in foods and human urine.^2,15,20−23^ However, these βCs did not arise from PHP-THβC-3-COOH. In a recent work, we provided the first evidence that the so-called carbohydrate-derived aromatic βCs came from 3-deoxyglucosone, an intermediate formed from glucose and particularly fructose.^15^ 3-Deoxyglucosone belongs to the group of α-dicarbonyl compounds that are reactive substances generated in foods and *in vivo*. Among them, the most important are glyoxal and methylglyoxal in addition to 3-deoxyglucosone. These compounds arise from the degradation of carbohydrates and form during glycation processes.^24−27^ Methylglyoxal also forms by alternative routes such as glycolysis from dihydroxyacetone (DHA) released during metabolism.^28^ These α-dicarbonyl compounds react with free amino acids and free amino groups of proteins affording advanced glycation end products (AGEs) that could play a role in human diseases such as diabetes and neurodegenerative and cardiovascular diseases.^24,25,29−33^ So far, adducts of lysine, arginine, and cysteine have been described.^34^ In this regard, the current research was aimed to investigate possible new adducts and AGE products arising from α-dicarbonyl compounds (glyoxal, methylglyoxal, and 3-deoxyglucosone) and tryptophan and, subsequently, to determine the factors and mechanisms influencing the formation of these compounds as well as their presence in foods. As a result, this highlights the formation of new α-dicarbonyl-derived β-carboline alkaloids produced from tryptophan and α-dicarbonyl compounds (glyoxal, methylglyoxal, and 3-deoxyglucosone) as well as their occurrence and formation in foods and food processing.

## Materials and Methods

### Chemical Compounds and Foods

**Figure 1:**
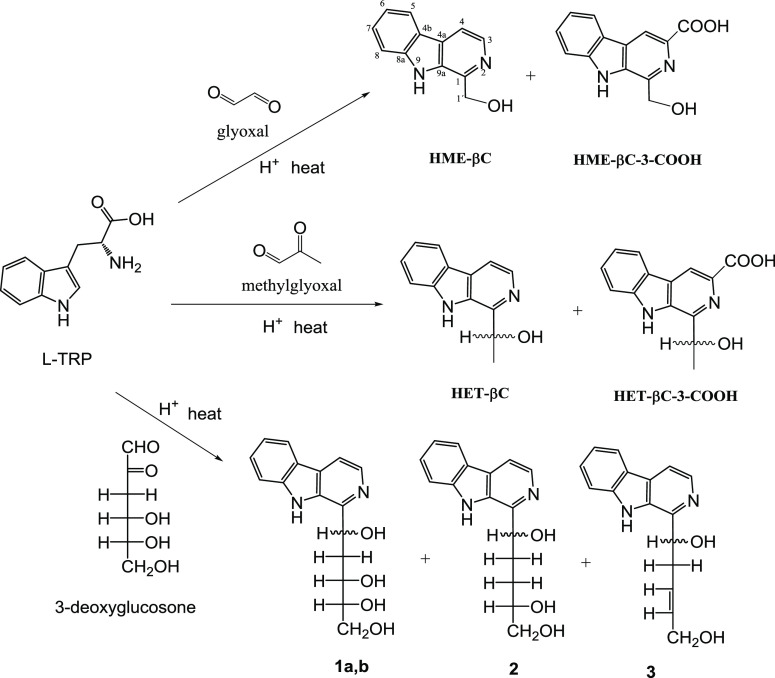
l-Tryptophan reacts with the α-dicarbonyl (1,2-dicarbonyl) compounds glyoxal, methylglyoxal, and 3-deoxyglucosone, affording α-dicarbonyl-derived β-carboline compounds.

Commercial samples of foods (Table 1) were purchased locally and from the internet and were processed and analyzed as indicated below. l-Tryptophan, glyoxal (40% in water), and methylglyoxal (40% in water) were obtained from Sigma-Aldrich (Saint Louis, MO, USA). d-(+)-Glucose monohydrate was obtained from Merck (Darmstadt, Germany), d-(−)-fructose from Sigma-Aldrich, and 3-deoxy-d-glucosone from Biosynth-Carbosynth (Compton, Newbury, UK). The β-carbolines derived from the reaction of the α-dicarbonyl compounds glyoxal and methylglyoxal (Figure 1) with tryptophan were prepared and characterized as follows:

**Table 1:** Concentrations of HET-βC Determined in Food Samples^a^

foods	*n*	*x* (ng/g)	SD	range
fried tomato	3	23.3	8.1	16.8–32.4
concentrated tomato	4	84.5	28.8	57.8–114.4
ketchup	3	20.4	5.4	15.5–26.2
tomato juice	4	23.6	21.9	Nd–44.8
dried tomato	1	309		
fried onion	6	112.7	108.7	10.8–232.2
dried fruit	11	20.4	35.3	Nd–123
jam	6	18.7	21.7	Nd–44.0
cereals	10	23.6	20.5	Nd–68.9
cereal bar	3	18.2	14.6	5.1–33.9
cookies	17	20.6	13.44	Nd–45.5
toasted/fried bread	4	66.3	29.4	23.8–91.5
bread	3	19.5	17.6	Nd–34.3
sugar cane molasses	1	256.8		
toasted beer	3	17.7	2.0	16–19.9
manuka honey	3	127	61.6	40.4–183.4
floral honey	3	13.0	3	10.3–16.6

a Nd: not detected.

#### 1-Hydroxymethyl-β-carboline (9*H*-Pyrido[3,4-*b*]indol-1-yl)methanol) (HME-βC)

l-Tryptophan (0.9 mmol) dissolved in phosphate buffer (pH 3) was reacted with glyoxal (1.8 mmol) at 80–90 °C for 12 h. The crude of the reaction mixture was filtered, and the filtrate was adjusted to pH 8–9 with 0.1 M NaOH and extracted with dichloromethane, which was evaporated to obtain the compound as a solid (18 mg) (10%). Spectral characterization was accomplished by ^1^H-NMR, ^13^C-NMR, COSY, TOCSY, HSQC, and HMBC experiments (Supporting Information): ^1^H NMR (400.13 MHz, DMSO) δ 11.36 (s, 1H), 8.24 (d, *J* = 5.1 Hz, 1H), 8.21 (d, *J* = 8.0 Hz, 1H), 8.01 (d, *J* = 5.1 Hz, 1H), 7.66 (d, *J* = 8.3 Hz, 1H), 7.52 (dd, *J* = 8.3 Hz, *J* = 7.5 Hz, 1H), 7.22 (dd, *J* = 8.0 Hz, *J* = 7.5 Hz, 1H), 4.96 (s, 2H). ^13^C NMR (100.62 MHz, DMSO) δ 144.93, 140.53, 136.85, 133.46, 127.92, 127.88, 121.51, 120.54, 119.11, 113.75, 112.24, 63.53. HR-MS (Agilent 6200 Series Q-TOF): found (M + H)^+^*m*/*z* 199.0858. Calculated for (C_12_H_10_N_2_O) + H^+^*m*/z 199.0866. Purity was higher than 95% by HPLC-DAD.

#### 1-(1-Hydroxyethyl)-β-carboline (1-(9*H*-Pyrido[3,4-*b*]indol-1-yl)ethan-1-ol) (HET-βC)

l-Tryptophan (0.9 mmol) dissolved in phosphate buffer (pH 2–3) was reacted with methylglyoxal (1.2 mmol) at 80–90 °C for 15 h. The crude of the reaction mixture was filtered, and the filtrate was adjusted to pH 8–9 with 0.1 M NaOH and extracted with dichloromethane. The organic phase was extracted with an aqueous solution (pH 3), and this aqueous phase was adjusted to pH 8–9 and extracted with dichloromethane and evaporated to obtain the compound as a solid (31.7 mg) (16.6%). Spectral characterization was accomplished by H-NMR, ^13^C-NMR, COSY, TOCSY, HSQC, and HMBC experiments (Supporting Information). ^1^H NMR (400.13 MHz, DMSO) δ^1^H: 11.23 (s, 1H), 8.23 (d, *J* = 4.6 Hz, 1H), 8.19 (d, *J* = 7.6 Hz, 1H), 7.99 (d, *J* = 4.6 Hz, 1H), 7.69 (d, *J* = 8.3 Hz, 1H), 7.51 (dd, J = 8.3 Hz, *J* = 7.7 Hz, 1H), 7.21 (dd, *J* = 7.6 Hz, *J* = 7.7 Hz, 1H), 5.68 (d, *J* = 3.6 Hz, 1H), 5.20 (m, 1H), 1.55 (d, *J* = 6.6 Hz, 3H). ^13^C NMR (100.62 MHz, DMSO) δ^13^C: 148.68, 140.49, 136.60, 132.26, 128.19, 127.77, 121.35, 120.44, 118.99, 113.47, 112.38, 69.32, 22.89. HR-MS (Agilent 6200 Series Q-TOF): found (M + H)^+^*m*/*z* 213.1019. Calculated for (C_13_H_12_N_2_O) + H^+^*m*/*z* 213.1023. Purity was higher than 95% by HPLC-DAD.

#### 1-Hydroxymethyl-β-carboline-3-carboxylic Acid (1-(Hydroxymethyl)-9*H*-pyrido[3,4-*b*]indole-3-carboxylic Acid) (HME-βC-3-COOH)

l-Tryptophan (0.9 mmol) dissolved in phosphate buffer (pH 1.4) was reacted with glyoxal (1.5 mmol) at 90–100 °C for 18 h. The crude of the reaction mixture was filtered, and the filtrate was adjusted to pH 9 with 2 N NaOH and washed with dichloromethane. The aqueous phase was acidified, concentrated in a rotary evaporator, loaded into a column chromatography containing C18 sorbent, and eluted with 0.5% (v/v) formic acid in water with increased percentages of acetonitrile (0–50%). The compound was eluted with 5% of acetonitrile in 0.5% formic acid and evaporated to obtain the product (5.0 mg) (2.3%). Spectral characterization was accomplished by ^1^H-NMR, ^13^C-NMR, COSY, TOCSY, HSQC, and HMBC experiments (Supporting Information). ^1^H NMR (400.13 MHz, DMSO) δ: 11.89 (s, 1H), 8.84 (s, 1H), 8.37 (d, *J* = 7.8 Hz, 1H), 7.71 (d, *J* = 8.4 Hz, 1H), 7.59 (dd, *J* = 8.2 Hz, *J* = 7.1 Hz, 1H), 7.30 (dd, *J* = 7.8 Hz, *J* = 7.2 Hz, 1H), 5.03 (s, 2H). ^13^C NMR (100.62 MHz, DMSO) δ: 167.15, 144.74, 141.53, 135.32, 133.38, 129.41, 128.98, 122.48, 121.37, 120.59, 116.77, 113.08, 63.39. HR-MS (Agilent 6200 Series Q-TOF): found (M + H)^+^ m/z 243.0763. Calculated for (C_13_H_10_N_2_O_3_) + H^+^: *m*/*z* 243.0764. Purity was higher than 95% by HPLC-DAD.

#### 1-(1-Hydroxyethyl)-β-carboline-3-carboxylic Acid (1-(Hydroxyethyl)-9*H*-pyrido[3,4-*b*]indole-3-carboxylic Acid) (HET-βC-3-COOH)

l-Tryptophan (0.9 mmol) dissolved in phosphate buffer (pH 1.4) was reacted with methylglyoxal (1.05 mmol) at 90–100 °C for 11 h. The crude of the reaction mixture was filtered, and the filtrate was adjusted to pH 9 with 2 N NaOH and washed with dichloromethane. The aqueous phase was then taken to pH 4–5, washed again with dichloromethane, then concentrated in a rotary evaporator, loaded into a column chromatography containing C18 sorbent, and eluted with 0.5% (v/v) formic acid in water with increased percentages of acetonitrile (0–50%). The compound was eluted with 5–10% of acetonitrile in 0.5% formic acid that was evaporated to obtain the product (6.83 mg) (3%). Spectral characterization was accomplished by ^1^H-NMR, ^13^C-NMR, COSY, TOCSY, HSQC, and HMBC experiments (Supporting Information). ^1^H NMR (400.13 MHz, DMSO) δ ^1^H: 11.77 (s, 1H), 8.81 (s, 1H), 8.36 (d, *J* = 7.9 Hz, 1H), 7.74 (d, *J* = 8.3 Hz, 1H), 7.58 (dd, *J* = 8.3, *J* = 7.6 Hz, 1H), 7.29 (dd, *J* = 7.9 Hz, *J* = 7.6 Hz, 1H), 5.28 (m, 1H), 1.57 (d, *J* = 6.6 Hz, 3H). ^13^C NMR (100.62 MHz, DMSO) δ ^13^C: 166.78, 147.97, 141.06, ca. 135, 133.65, 128.60, 128.41, 121.84, 120.82, 119.99, 115.94, 112.71, 68.73, 23.09. HR-MS (6200 Series Q-TOF): found (M + H)^+^*m*/*z* 257.0907. Calculated for (C_14_H_12_N_2_O_3_) + H^+^*m*/*z* 257.0921. Purity was higher than 95% by HPLC-DAD.

The carbohydrate-derived β-carbolines, 1-(1,3,4,5-tetrahydroxypent-1-yl)-β-carboline diastereoisomers (**1a/b**), 1-(1,4,5-trihydroxypent-1-yl)-β-carboline (**2**), and 1-(1,5-dihydroxypent-3-en-1-yl)-β-carboline (**3**) (Figure 1), were obtained from a reaction of glucose with l-tryptophan in high temperature and acidic media (pH 1) and isolated by column chromatography (C18) as previously.^20,22^ These compounds have been previously identified in foods, and their complete spectral data have been reported.^15,20,22,23,35^

### Formation of βCs Derived from α-Dicarbonyl Compounds in Model Reactions and Foods

Model reactions containing l-tryptophan and glyoxal, methylglyoxal, 3-deoxyglucosone, or the carbohydrates, glucose or fructose, were carried out to evaluate the formation of α-dicarbonyl-derived βCs. Solutions of l-tryptophan (0.5 mg/mL) and glyoxal (0.04 mg/mL), methylglyoxal (0.04 mg/mL), or 3-deoxyglucosone (0.1 mg/mL) in 100 mM phosphate buffer adjusted at different pHs (1.3, 3.1, 5, 7.4, and 9) were reacted in a water bath at 90 °C for 2–4 h and analyzed directly by HPLC. Solutions of l-tryptophan (0.5 mg/mL) and glyoxal (0.04 mg/mL), methylglyoxal (0.04 mg/mL), or 3-deoxyglucosone (0.1 mg/mL) in 100 mM phosphate buffer adjusted at pH 3.1 were reacted in glass tubes with ground-glass stoppers at different temperatures (25–110 °C) for 2–4 h and analyzed by HPLC. l-Tryptophan solutions (0.5 g/L) and glucose (5 g/L) or fructose (4.5 g/L) were reacted in buffer phosphate (pH 2.85) at 90 °C for 20 h and analyzed by HPLC. Also, solutions of l-tryptophan (0.5 mg/mL) and glucose (5 mg/mL) or fructose (4.5 mg/mL) in 100 mM phosphate buffer (pH 2.85) were reacted at different temperatures (90–130 °C) for 2 h whereas solutions of l-tryptophan (0.5 mg/mL) and fructose (4.5 mg/mL) in different phosphate buffers (pH 3, 5, and 7.4) were reacted at 130 °C for 2 h. Aliquots of the reactions were injected into the RP-HPLC and analyzed by DAD, fluorescence, and HPLC-MS. All reactions were carried out at least in duplicate. To study formation in foods, natural tomato puree (2 g) was heated in an oven (90 °C, 5 h) and tomato cherry was heated in an oven until dried (80 °C, 12.5 h). These samples were analyzed after extraction by SPE.

### Isolation of α-Dicarbonyl-Derived β-Carbolines in Foods by Solid Phase Extraction (SPE)

The α-dicarbonyl-derived βCs were isolated from foods by SPE using propylsulfonic acid-derivatized silica PRS columns (Bond Elut, 500 mg, 3 mL size, Agilent). Samples of foods (2–5 g) were added with 0.6 M HClO_4_ (15–20 mL), homogenized using an ULTRA-TURRAX homogenizer, and centrifuged at 10,000 rpm, 15 min at 0–5 °C. The conditioning of PRS columns was made with methanol and 0.1 M HCl. Aliquots (5 mL) were spiked with 0.5 mL of 1-ethyl-β-carboline (EβC) solution (0.2 mg/L) used as an internal standard (IS) and subsequently loaded onto PRS columns using a vacuum manifold. After washing with deionized water (2 mL) and 0.4 M K_2_HPO_4_ (pH 9.1) (3 mL), the βCs were eluted with 3 mL of 0.4 M K_2_HPO_4_ (pH 9.1):methanol (1:1) and analyzed by HPLC-fluorescence whereas the presence of compounds was confirmed by HPLC-MS. The performance of the SPE procedure gave recoveries of 85 and 94% (*n* = 3) and repeatabilities (RSD) of 3 and 2% for HME-βC and HET-βC (100 μg/L), respectively*.*

### Chromatographic Analysis of βCs and Identification by HPLC-MS.

The chromatographic analysis of the α-dicarbonyl-derived βCs from both synthetic and model reactions was performed using an Agilent HPLC 1050 with a 1100 series DAD and a 1046A fluorescence detector. The analysis of the α-dicarbonyl-derived βCs isolated from foods was carried out with an Agilent HPLC 1200 series with a 1200 series DAD and a 1260 series fluorescence detector (Agilent). A 150 mm × 3.9 mm, 5 μm, Novapak C18 column (Waters) was used for HPLC separation. Eluents: 50 mM ammonium phosphate buffer adjusted to pH 3 with phosphoric acid (eluent A); 20% of eluent A in acetonitrile (eluent B). The gradient was 0–32% B in 8 min, then 90% B at 18 min, and 100% B at 20 min. The flow rate was 1 mL/min, the oven temperature was 40 °C, and the injection volume was 20 μL. Detection was carried out with absorbance (DAD) and fluorescence (300 nm excitation/433 nm emission). Quantitative analyses of βCs in model reactions were done with calibration curves of standards with absorbance detection at 254 nm for βCs and 280 nm for βC-3-carboxylic acid. The α-dicarbonyl-derived βCs isolated by SPE in foods were detected by fluorescence at 300 nm (excitation) and 433 nm (emission). Quantitative analysis was obtained from calibration curves of standard solutions of known concentration of HET-βC against EβC used as an internal standard (IS) and carried out through the entire SPE isolation procedure. HME-βC was determined in some samples by HPLC-MS (*m*/*z* 199) following identification by MS. Identification of compounds was carried out by DAD and fluorescence spectra of the chromatographic peaks, coelution with authentic standards, and HPLC-MS. Model reactions and the SPE food extracts were analyzed by HPLC-MS to confirm the identity of compounds. SPE extracts were concentrated using a vacuum concentrator and analyzed by HPLC-MS. The instrument used for βC identification in foods and model reactions was an HPLC-MS Waters separation module Alliance e2695 fitted with a quadrupole QDa Acquity and a Waters Photodiode Array Detector (PDA) 2996, working under positive electrospray ionization mode (ESI+) and equipped with a 2.1 × 100 mm, 3 μm, 100 Å, C18 Atlantis T3 column (Waters). Chromatographic separation was accomplished with a program containing the eluents A (water), B (ACN), and C (2% formic acid) under a gradient from 5% B, 5% C, and 90% A to 90% B, 5% C, and 5% A in 18 min. The flow rate was 0.350 mL/min, and injection volume was 9 μL. The mass spectra were acquired under ESI positive ion ionization mode at various cone voltages (10, 20, and 40 V) with a mass range of 85–1250 *amu*.

### Chiral Chromatography of βCs

The βC HET-βC (Figure 1) contains a chiral center at C-1′. A separation of the enantiomers of the synthetized HET-βC and HET-βC isolated from foods was accomplished with an HPLC 1200 series (Agilent) by chiral chromatography using a 2.1 × 150 mm, 5 μm, Chiralpak IA column working under isocratic conditions: water (35%) and methanol (65%) with a flow rate of 0.2 mL/min and temperature of 30 °C. The βC compounds were detected by DAD and fluorescence (240 nm excitation/433 nm emission). The βCs isolated from foods by SPE were extracted with dichloromethane, concentrated to dryness, redissolved in phosphate buffer (pH 9.1):methanol (1:1), and injected into the chiral column. Also, the chromatographic fraction corresponding to HET-βC was isolated by RP-HPLC by collecting the compound at the end of the detector and injected into the chiral column.

## Results

### β-Carbolines Derived from α-Dicarbonyl Compounds

**Figure 2:**
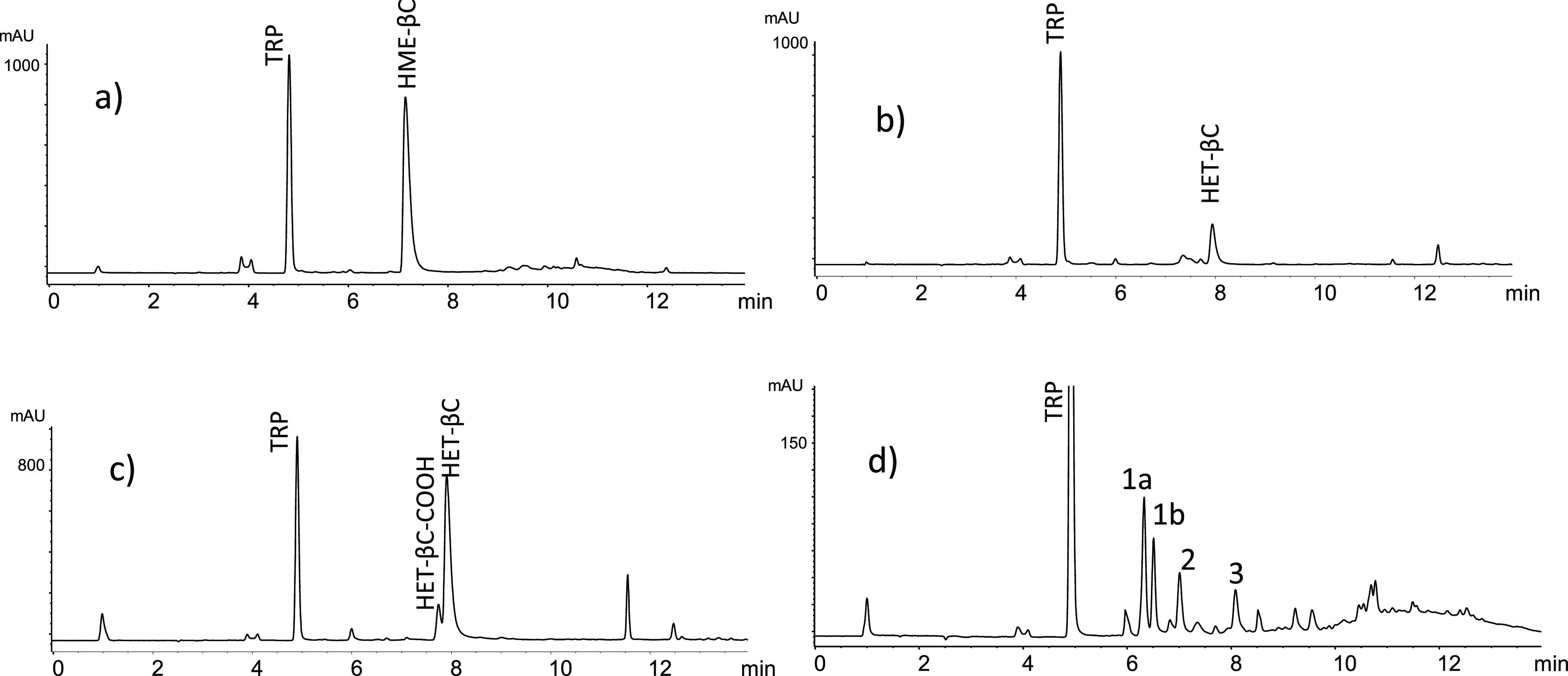
RP-HPLC chromatograms of β-carbolines formed in the reactions of l-tryptophan with glyoxal (pH 3.1, 90 °C, 2 h) (a), methylglyoxal (pH 3.1, 90 °C, 2 h) (b), methylglyoxal (pH 1.3, 90 °C, 2 h) (c), and 3-deoxyglucosone (pH 3.1, 110 °C, 2 h) (d).

**Figure 3:**
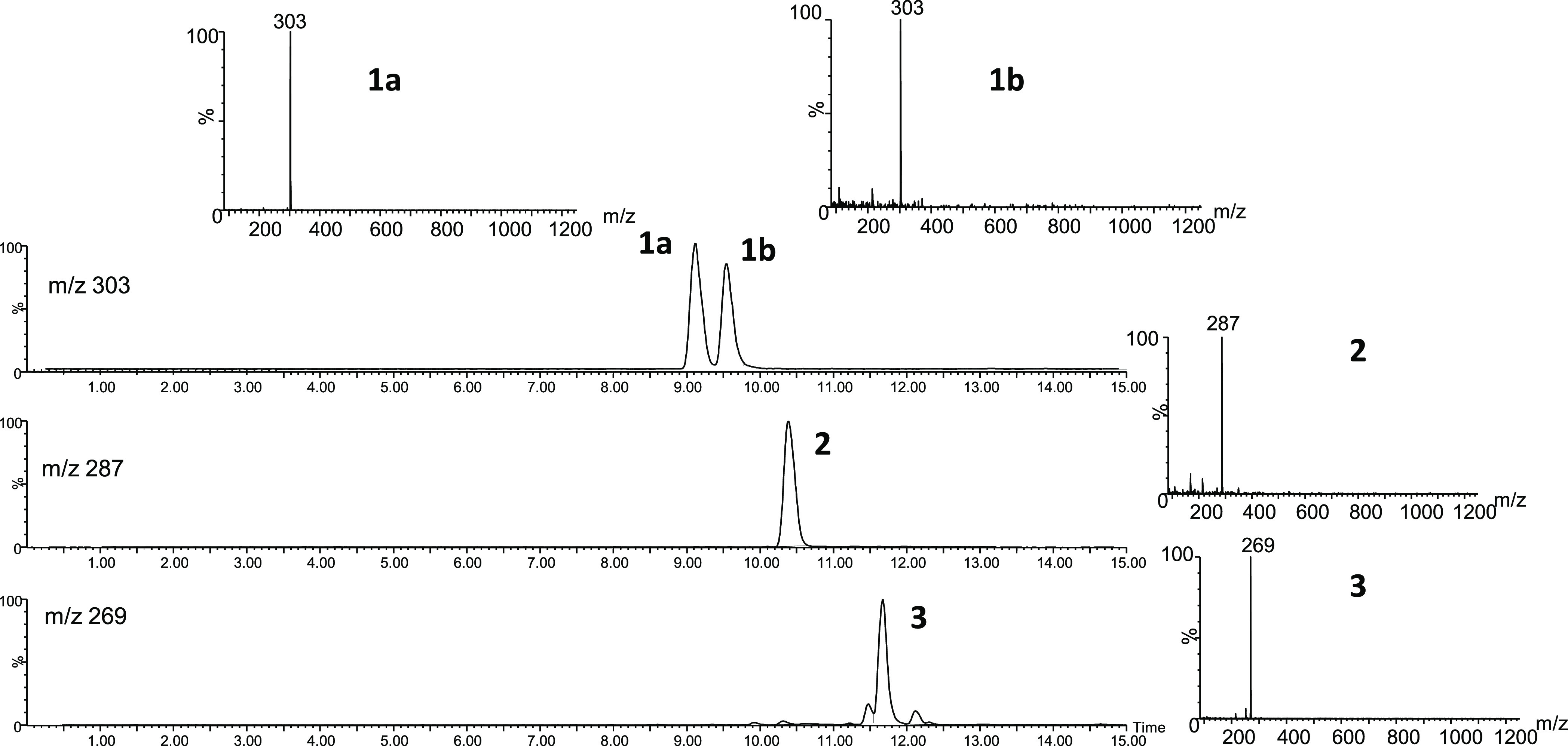
HPLC-MS (ESI-positive ionization, 20 V) of βCs **1**–**3** identified in the reaction of 3-deoxyglucosone (0.1 mg/mL) with l-tryptophan (0.5 mg/mL) (pH 3.1, 110 °C, 2 h). The spectra show the (M + H)^+^ ions, but higher fragmentation is produced at higher fragmentation voltages.^15^

**Figure 4:**
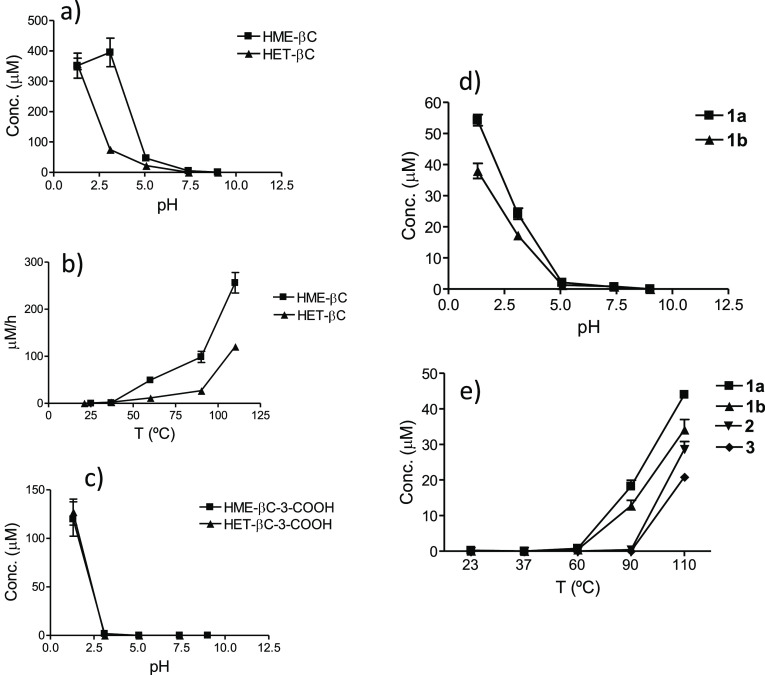
Formation of α-dicarbonyl-derived β-carbolines from l-tryptophan (0.5 mg/mL) and glyoxal (0.04 mg/mL) (HME-βC and HME-βC-3-COOH) or methylglyoxal (0.04 mg/mL) (HET-βC and HET-βC-3-COOH) as a function of pH (90 °C, 4 h) (a, c) and temperature (pH 3.1, 2 h) (b). Formation of βCs **1**–**3** from l-tryptophan (0.5 mg/mL) and 3-deoxyglucosone (0.1 mg/mL) as a function of pH (90 °C, 4 h) (d) and temperature (pH 3.1, 2 h) (e).

Model reactions showed that l-tryptophan reacted with the α-dicarbonyl (1,2-dicarbonyl) compounds, glyoxal, and methylglyoxal, resulting in new β-carbolines (Figures 1 and 2). These compounds were synthetized and characterized by NMR and MS (see above and the Supporting Information, Figures S1–S5). Glyoxal afforded 1-hydroxymethyl-β-carboline (HME-βC), and methylglyoxal afforded 1-(1-hydroxyethyl)-β-carboline (HET-βC). In addition, l-tryptophan reacted with the α-dicarbonyl compound 3-deoxyglucosone, giving the so-called carbohydrate-derived βCs studied in foods:^15,20,22,35^ 1-(1,3,4,5-tetrahydroxypent-1-yl)-β-carboline isomers (**1a/b**), 1-(1,4,5-trihydroxypent-1-yl)-β-carboline (**2**), and 1-(1,5-dihydroxypent-3-en-1-yl)-β-carboline (**3**) (Figures 1–3). The formation of β-carbolines from glyoxal and methylglyoxal highly increased with acidic pH and upon increasing temperature (Figure 4a–c). Moderate temperatures were needed to result in some product formation, and a very low amount was formed at room temperature or under physiological conditions. The formation of βCs **1**–**3** arising from 3-deoxyglucosone also increased with acidic pH and with increasing temperature (Figure 4d,e). Relative formation of βCs **2** and **3** vs **1a/b** was favored by increasing temperature.

**Figure 5:**
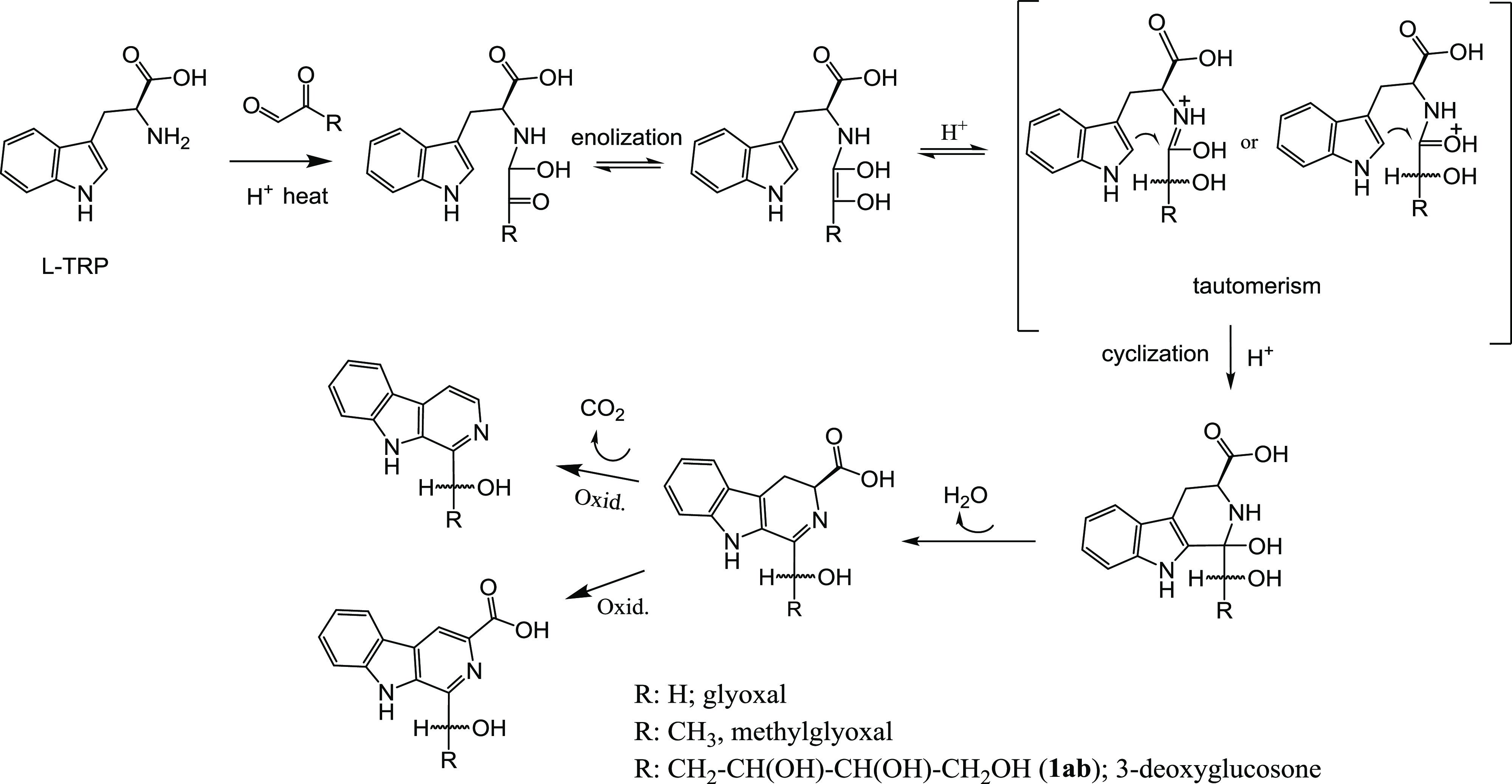
Proposed mechanism for the formation of α-dicarbonyl-derived β-carbolines from glyoxal, methylglyoxal or 3-deoxyglucosone, and l-tryptophan.

**Figure 6:**
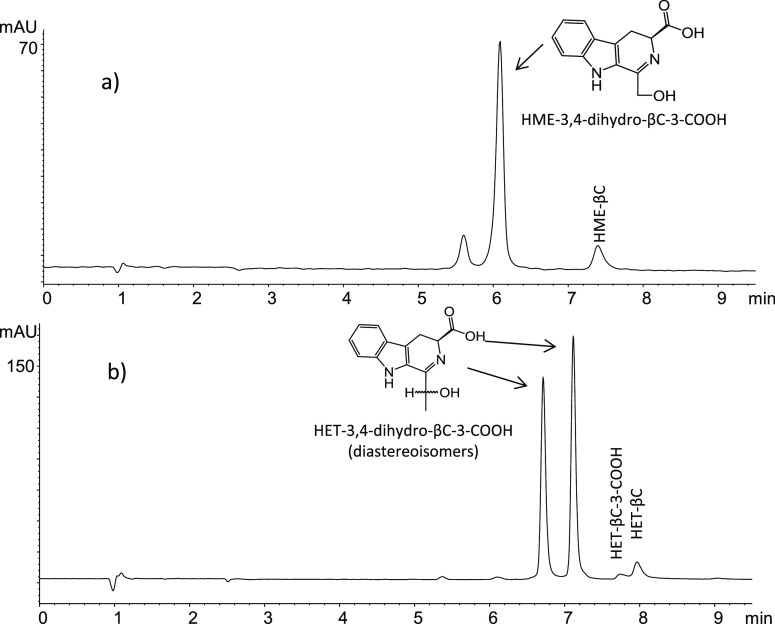
RP-HPLC chromatogram (absorbance at 355 nm) with the 3,4-dihydro-β-carboline-3-carboxylic acid intermediates formed in the reaction of l-tryptophan (0.5 mg/mL) and glyoxal (0.04 mg/mL) (70 °C, pH 3, 30 min) (a) or methylglyoxal (0.04 mg/mL) (60 °C, pH 1.3, 2 h) (b) that afforded HME-βC and HME-βC-3-COOH or HET-βC and HET-βC-3-COOH. The corresponding fully aromatic βCs that are the final products increased during reaction time, and their response is higher at 254 (βC) or 280 nm (βC-COOH).

The carbonyl group (C=O) in the α-dicarbonyl is converted into an alcohol (C-OH) substituent in these βCs. A mechanism for this is proposed in Figure 5. l-Tryptophan reacts with the α-dicarbonyl compound that could follow an imine-enamine or keto-endiol tautomerism with cyclization to give the corresponding 3,4-dihydro-β-carboline-3-carboxylic acid. A subsequent oxidation with the loss of the carboxylic group affords the aromatic β-carboline. Two types of evidence were obtained here supporting this sequence. First, the corresponding 3,4-dihydro-β-carboline-3-carboxylic acid compounds were detected and identified as intermediates (Figure 6) before disappearing to give the corresponding aromatic β-carbolines. Thus, the reaction of l-tryptophan with glyoxal gave 1-hydroxymethyl-3,4-dihydro-β-carboline-3-carboxylic acid (DAD, λmax at 355 nm; MS: *m*/*z* at 245 (M + H)^+^ and fragments 199, 181, and 169) whereas the reaction of l-tryptophan with methylglyoxal gave 1-(1-hydroxyethyl)-3,4-dihydro-β-carboline-3-carboxylic acid as two diastereoisomers (chiral centers at C-1′ and C-3) (DAD, λmax at 355 nm; MS at *m*/*z* 259 (M + H)^+^, and fragments 213, 195, 186, and 169). These dihydro-β-carbolines were isolated at the exit of the RP-HPLC column, and following heating (90 °C), they converted into the corresponding fully aromatic βC, as determined by HPLC-DAD-MS. The second evidence is that the corresponding fully aromatic β-carboline-3-carboxylic acids (βC-3-COOH) were formed as important secondary products along with the main βCs in reactions at low pH (pH 1.3) (Figures 1, 4c, and 5), supporting that the oxidation to the fully aromatic βCs occurred at the end of the process and it occurred without decarboxylation under these conditions. These βC-3-COOHs were isolated and characterized by NMR and MS (Materials and Methods section and Figures S3 and S4).

### α-Dicarbonyl-Derived βCs Occurred in Reactions of Tryptophan and Carbohydrates

**Figure 7:**
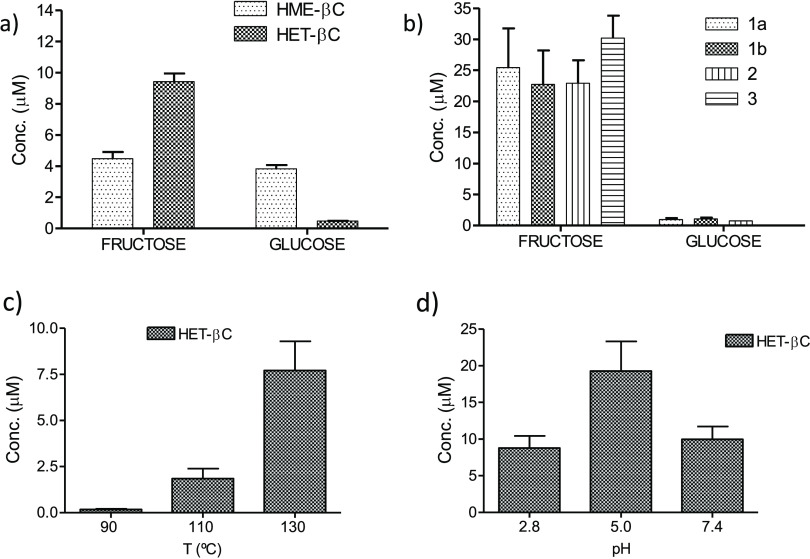
α-Dicarbonyl-derived β-carbolines HME-βC, HET-βC, and βCs **1**–**3** formed in reactions of l-tryptophan (0.5 mg/mL) with glucose (5 mg/mL) or fructose (4.6 mg/mL) (90 °C, pH 2.8, 20 h) (a, b). Formation of HET-βC in the reaction of l-tryptophan (0.5 mg/mL) and fructose (4.6 mg/mL) at different temperatures (pH 2.8, 2 h) (c) and pHs (130 °C, 2 h) (d).

α-Dicarbonyl-derived βCs were found in the reactions of l-tryptophan with carbohydrates. Both HME-βC and HET-βC occurred in model reactions of l-tryptophan with fructose or glucose incubated under heating in acidic conditions as confirmed by HPLC-MS (Figure S6). The carbohydrate-derived βCs **1**–**3** arising from 3-deoxyglucosone were also formed in those reactions as reported here and in a previous work.^15^ The βCs derived from methylglyoxal (HET-βC) and glyoxal (HME-βC) were formed in lower amounts than the βCs **1**–**3** arising from 3-deoxyglucosone (Figure 7a,b). Fructose gave higher amounts of HET-βC and βCs **1**–**3** than glucose (Figure 7a,b). The amounts of HET-βC and HME-βC (not shown) increased with temperature (Figure 7c). Remarkably, HET-βC was formed in model reactions of l-tryptophan and fructose at high temperature (110–130 °C) and higher pH 5–7.4 (Figure 7d) as the main βC. Moreover, HET-βC was also formed from 3-deoxyglucosone and l-tryptophan at high temperature (110–130 °C) (Figure S7) in addition to βCs **1**–**3** (ca. of 7% of βCs **1**–**3** at 130 °C, pH 3, 2 h).

### Occurrence of α-Dicarbonyl-Derived βCs in Foods

**Figure 8:**
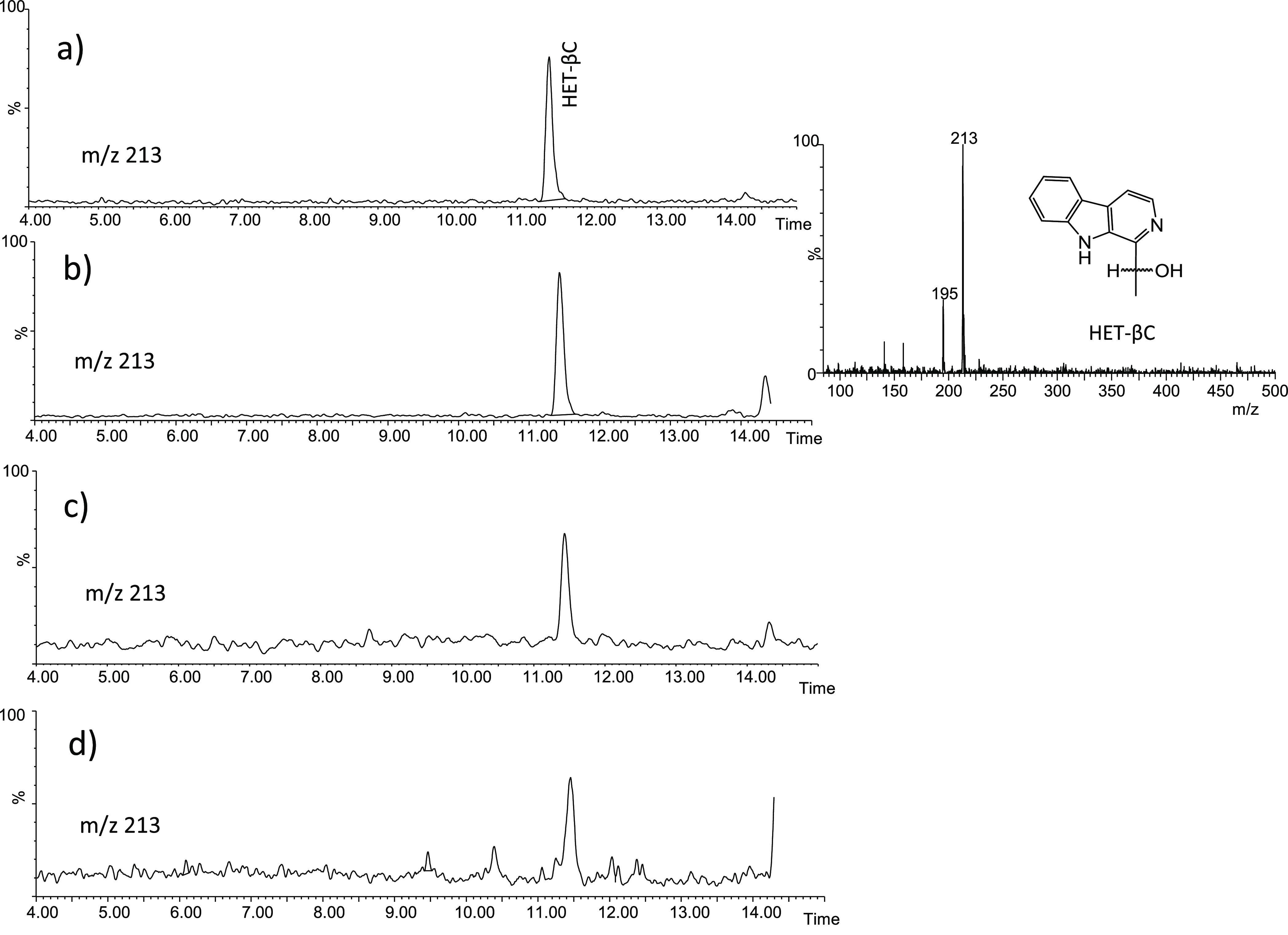
Identification of HET-βC in food extracts by HPLC-MS analysis (ESI positive ionization, 20 V): crispy fried onion (a), Manuka honey (b), crunchy dried tomato (c), and toasted bread (d).

**Figure 9:**
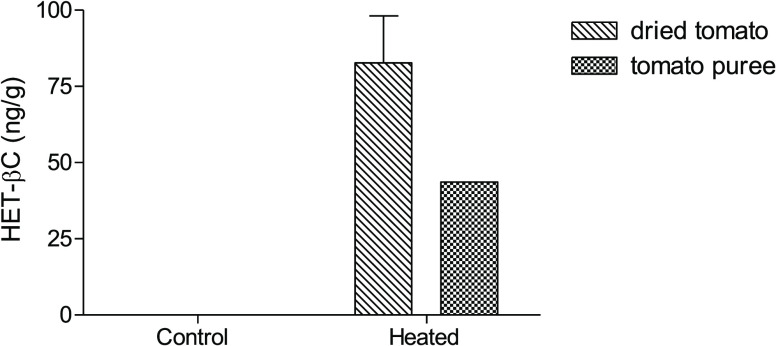
Formation of HET-βC in tomato puree heated in an oven (90 °C, 5 h) and in tomato cherry dried in an oven (80 °C, 12.5 h). The control samples before heating did not contain HET-βC.

The presence of α-dicarbonyl-derived βCs in foods was investigated. The βCs **1**–**3** arising from 3-deoxyglucosone were studied in a previous work.^15^ In this work, the βC HET-βC was identified by HPLC-MS (*m*/*z* at 213 (M + H)^+^ and 195 (213–18)) (Figure 8) and it appeared in many processed foods. Analysis was accomplished by HPLC with fluorescence detection (Figure S8), and the content ranged from undetected to hundreds of ng/g (Table 1). This occurred in processed tomato products (dried tomato, tomato concentrate, fried tomato, ketchup sauce, and tomato juice), vegetable and fruit products (*e.g.*, fried onion), toasted/fried bread, cookies, cereals, sugar cane molasses, and honey. The highest levels were found in dried tomato (309 ng/g), sugar cane molasses (257 ng/g), Manuka honey (127 ng/g), and fried onion (113 ng/g). HET-βC was formed during the heating process as seen for dried tomatoes and tomato puree (Figure 9). Compared with HET-βC, HME-βC was undetectable in most foods or instead appeared in very low amounts. It was detected by HPLC-MS (Figure S9) (at *m*/*z* 199 (M + H)^+^ and 181 (199–18)) in dried tomato (80.5 ng/g), fried onion (46.3 ng/g), tomato concentrate (15.4 ng/g), ketchup (16 ng/g), and cereals (28 ng/g).

Finally, HET-βC contains a chiral center at C-1′ with two possible enantiomers. It was analyzed by chiral chromatography using the tris(3,5-dimethylphenylcarbamate) derivative of amylose as an immobilized chiral selector (Chiralpak IA) that allowed the enantiomeric resolution. The results obtained indicated that the synthetized HET-βC was present as a racemic mixture, and the compound HET-βC isolated from foods (*e.g.*, Manuka honey and others) also appeared as a racemic mixture (Figure S10). These results evidence that HET-βC occurs in foods following the same chemical reaction.

## Discussion

The results reported above show that the α-dicarbonyl compounds, glyoxal, methylglyoxal, and 3-deoxyglucosone react with tryptophan to give β-carbolines. The reactions of glyoxal and methylglyoxal afforded the new β-carbolines, HME-βC and HET-βC, respectively, as well as their 3-carboxylic acids, whereas 3-deoxyglucosone gave rise to the β-carbolines **1a/b-3**. The first evidence of this reaction was obtained while studying the carbohydrate-derived βCs **1**–**3** in foods and model reactions.^15^ As shown here, the α-dicarbonyl-derived β-carbolines increased under acidic conditions and with increasing temperature. However, they can also form at pH 5–7 at high temperature (*e.g.*, 110 °C and higher) as seen with HET-βC. Under room temperature and physiological conditions (37 °C and pH 7.4), the formation of these βCs was not favored. However, they formed during heating of foods and in the reactions of carbohydrates with tryptophan. The generation of these compounds during food heating could be remarkable. We know that carbonyl compounds occurring in foods such as formaldehyde and acetaldehyde afford 1,2,3,4-tetrahydro-β-caboline-3-carboxylic acid (THβC-3-COOH) through a Pictet–Spengler reaction with tryptophan.^12,36^ These tetrahydro-β-carbolines are direct precursors of aromatic βCs such as norharman and harman after oxidative decarboxylation in a chemical or enzymatic process.^8,18,19,37^ However, the mechanism to afford the α-dicarbonyl-derived βCs reported here differs. It requires the conversion of the carbonyl (C=O) at C-2′ of the α-dicarbonyl into an alcohol (−OH) substituent. This kind of conversion occurs in Maillard processes such as the formation of amide advanced glycation end products.^38,39^ A mechanism is proposed in Figure 5 in which the α-dicarbonyl compound reacts with l-tryptophan and follows an imine-enamine or keto-endiol tautomerism that cyclizes to give a 3,4-dihydro-β-carboline-3-carboxylic acid derivative intermediate that eventually affords the fully aromatic β-carboline through oxidative decarboxylation or alternatively the β-carboline-3-carboxylic acid with oxidation but without decarboxylation. This is supported with the detection of corresponding 3,4-dihydro-β-carboline-3-carboxylic acid intermediates at short reaction times that were isolated and converted by heating into the corresponding α-dicarbonyl-derived βCs. Moreover, the corresponding β-carboline-3-carboxylic acids were identified and characterized as important secondary products along with the main βC products in reactions at pH 1.3, suggesting that, in very acidic conditions, the oxidation to the fully aromatic βCs occurred without decarboxylation (Figure 5). This mechanism, proposed also for the formation of carbohydrate-derived βCs **1**–**3** in foods,^15^ differs from that of the Pictet–Spengler reaction because it leads to 3,4-dihydro-β-carboline-3-carboxylic acids and provides a rationalization for the OH substituent in the side chain of the β-carboline. Alternatively, methylglyoxal could afford 1-acetyl-β-carboline derivatives under some conditions common in microbial biotransformations and in marine organisms.^40−43^

The βCs derived from α-dicarbonyls appeared in reactions of carbohydrates with tryptophan incubated under heating. The βCs derived from methylglyoxal and 3-deoxyglucosone formed in higher amounts from fructose than glucose, whereas the βC derived from glyoxal resulted similarly from both glucose and fructose. 3-Deoxyglucosone, a main α-dicarbonyl intermediate from dehydration of carbohydrates and particularly fructose,^44^ is the precursor of βCs **1**–**3**.^15^ Carbohydrates undergo retroaldol-type cleavage during degradation by heating and afford aldehydes including glyoxal and methylglyoxal.^45^ Methylglyoxal is formed by retroaldol fragmentation of 3-deoxyglucosone, while glyoxal arises from degradation of glucose by retroaldol reactions and oxidation.^24^ Then, those α-dicarbonyl compounds generated during degradation of sugars react with tryptophan affording βCs. As seen here, at a temperature of 90 °C, the βCs **1**–**3** coming from 3-deoxyglucosone were higher than HET-βCs from methylglyoxal whereas HME-βC from glyoxal was present in the lowest amount (Figure 7). These results agree well with the relative levels of α-dicarbonyls generated from carbohydrates: 3-deoxyglucosone > methylglyoxal ≫ glyoxal.^28,44^ However, α-dicarbonyl levels could vary with temperature and pH^46^ and as a result affect the βCs produced. Thus, HET-βC was produced as the main βC from fructose and tryptophan at high temperature (110 °C and higher) and pH 5–7. HET-βC was also produced from 3-deoxyglucosone. This is probably due to an increased formation of methylglyoxal under these conditions. Therefore, the formation of HET-βC during heating (*e.g.*, cooking) in high temperature could be remarkable.

α-Dicarbonyls occur in foods as a result of the degradation of carbohydrates or from other routes. Low levels of glyoxal, ranging from 0.23 to 2.66 μg/mL, were found in coffee, barley coffee, and soy sauce,^47^ whereas methylglyoxal appeared in beverages with the highest amount in coffee (up to 25 μg/g).^48^ Glyoxal and methylglyoxal have been reported in cookies ranging from 4.8 to 26.0 and 3.7 to 81.4 mg/kg, respectively.^49^ 3-Deoxyglucosone was the predominant 1,2-dicarbonyl compound in foods with concentrations up to 410 mg/L in fruit juices, 2622 mg/L in balsamic vinegars, and 385 mg/kg in cookies.^28^ Relatively high levels of methylglyoxal have been found in Manuka honey, reaching up to 750 mg/kg.^28^ 1,2-Dicarbonyl compounds (α-oxoaldehydes) have been also reported in biological samples such as blood and plasma.^29,50^ These compounds are cytotoxic and might induce cellular damage.^30^ They react with free amino groups of amino acids and proteins affording irreversible advanced glycation end products (AGEs), which may have a role in diseases such as diabetes mellitus, Alzheimer’s disease, and atherosclerosis.^25,26,31,51^ Their reaction with lysine, arginine, or cysteine affords α-dicarbonyl adducts in the early and advanced glycation process.^25,34^ In this regard, the results presented here evidence the formation of α-dicarbonyl-derived β-carbolines from a reaction with tryptophan. These compounds could be a new type of AGEs. Under physiological conditions, the formation of α-dicarbonyl-derived β-carbolines might be rather limited. In contrast, these βCs occur in foods and food processing or cooking. Therefore, they are ingested via foods and could get distributed in biological tissues and fluids similarly to other βCs.^2^ The βCs **1**–**3** derived from 3-deoxyglucosone were determined in foods.^15^ Here, HET-βC arising from methylglyoxal was identified and quantified in commercial foods. It appeared in processed tomatoes, vegetables, and fruit products as well as toasted bread, cookies, and honey with concentrations ranging from undetected to hundreds of ng/g. HME-βC only appeared in some foods and with much lower amounts. These βCs formed during food processing by heating as seen here with dried tomatoes and tomato puree. Then, foods containing tryptophan and carbohydrates (fructose and/or glucose) that are processed by heating will afford α-dicarbonyl-derived βCs. Those conditions are needed to generate α-dicarbonyls from carbohydrates and to afford β-carbolines through reaction with tryptophan. The relative presence of these compounds in foods correlates with the levels of α-dicarbonyls reported in literature (i.e., 3-deoxyglucosone > methylglyoxal ≫ glyoxal). Thus, βCs **1**–**3** that result from 3-deoxyglucosone in foods^15^ were generally found in higher amounts than HET-βC arising from methylglyoxal whereas HME-βC from glyoxal was very low or undetectable. The presence and formation of HET-βC in Manuka honey are an exception. Manuka honey contains high levels of naturally occurring methylglyoxal.^28,52^ It is not coming from sugar degradation (or 3-deoxyglucosone degradation) but appears during ripening from dihydroxyacetone (DHA) that arises during glycolysis, and it is present in high levels in the nectar of flowers used by bees to make honey.^53^ This βC can be formed from the reaction of methylglyoxal with tryptophan during honey ripening. In fact, this βC increased when Manuka honey was heated (not shown). The results obtained with chiral chromatography showed that HET-βC isolated from foods (*e.g.*, Manuka honey) occurred as a racemic mixture similarly to the synthetic product, evidencing that its formation in foods follows the same chemical reaction. This compound has also been isolated from the fungi *Cordyceps sinensis* as a racemic mixture of enantiomers.^54^

The βC alkaloids are bioactive compounds, and their occurrence in foods and *in vivo* is relevant. They interact with CNS receptors, inhibit enzymes (MAO and kinases), and exhibit anticancer, antimicrobial, and antioxidant actions.^3^ Aromatic βCs are co-mutagenic in the presence of aromatic amines and can be bioactivated to give neurotoxic *N*-methyl-β-carbolinium cations.^2,3^ The βCs inhibit MAO and exhibit antidepressant, neuroprotective, and neurogenesis effects. The βCs norharman and harman are good inhibitors of MAO,^8,55^ whereas the βCs **1**–**3** show poor activity.^15^ The βCs derived from α-dicarbonyls reported in this work can be considered as new AGEs derived from tryptophan. They are generated in foods and during food heating/cooking. These compounds are ingested during diet and could get into the body as it occurs with other βCs.^56,57^ The exposure to βCs from α-dicarbonyls (glyoxal, methylglyoxal, and 3-deoxyglucosone) could account for up to thousands of μg/person a day.

Taken together, this work has shown that α-dicarbonyls such as glyoxal, methylglyoxal, and 3-deoxyglucosone react with tryptophan to give new βCs with an OH group in C-1′ that were characterized. The mechanism of formation of these βCs may occur through an imine-enamine or keto-endiol tautomerism and cyclization with the formation of dihydro-β-carboline-3-carboxylic acid intermediates and further oxidation with or without decarboxylation to give the aromatic βCs. The formation of α-dicarbonyl-derived βCs was favored in acidic conditions and at increased temperatures. These βCs formed in reactions of the carbohydrates fructose and glucose with tryptophan owing to the release of α-dicarbonyl compounds from carbohydrate degradation. These compounds occur in foods and are formed during food processing by heating. The βCs **1**–**3** arising from 3-deoxyglucosone appear in many processed foods.^15,20,22^ Here, it is reported that HET-βC derived from methylglyoxal was present in many processed foods ranging from undetectable to hundreds of ng/g whereas HME-βC arising from glyoxal appeared only in low amounts in some foods. These βCs come from α-dicarbonyls released from carbohydrates. An exception is Manuka honey where HET-βC comes from methylglyoxal, which is naturally present in this honey. The βCs are bioactive alkaloids, and therefore, the occurrence of α-dicarbonyl-derived βCs in foods and *in vivo* could be relevant. These compounds may exhibit biological actions after being absorbed, while their formation as a new type of AGEs involves the capture of reactive α-dicarbonyl compounds.

## Supporting Information Available

Tables with signals and NMR spectra of compounds and the supplementary figures mentioned in the Results section (PDF)
