# Supplementary material for: Formation, Characterization, and Occurrence of β-Carboline Alkaloids Derived from α-Dicarbonyl Compounds and l-Tryptophan
Source: J Agric Food Chem. 2022 Jul 12;70(29):9143–53. doi: 10.1021/acs.jafc.2c03187 (PMC9335879; doi:10.1021/acs.jafc.2c03187)
Supplement: Supplementary file 1 — jf2c03187_si_001.pdf [file jf2c03187_si_001.pdf]

SUPPLEMENTARY MATERIAL

FORMATION, CHARACTERIZATION AND OCCURRENCE OF β-CARBOLINE ALKALOIDS DERIVED FROM α-DICARBONYL COMPOUNDS AND *L*-TRYPTOPHAN

Tomás Herraiz[1]*, Adriana Peña[1], Haroll Mateo[1], Marta Herraiz[1], Antonio Salgado[2]

[1]Instituto de Ciencia y Tecnología de Alimentos y Nutrición (ICTAN-CSIC). Spanish National Research Council (CSIC). José Antonio Novais 10, Ciudad Universitaria 28040, Madrid (Spain).

[2]Centro de Espectroscopia de Resonancia Magnética Nuclear (CERMN). Universidad de Alcalá (UAH), Campus Universitario, Ctra. Madrid-Barcelona km 33.6, 28805, Alcalá de Henares, Madrid (Spain)

1) Structural characterization of α-dicarbonyl-derived βC compounds

1) 1-Hydroxymethyl-1-β-carboline; ((9*H*-pyrido[3,4-*b*]indol-1-yl)methanol); (comp. HME-βC; Comp. HA-5):

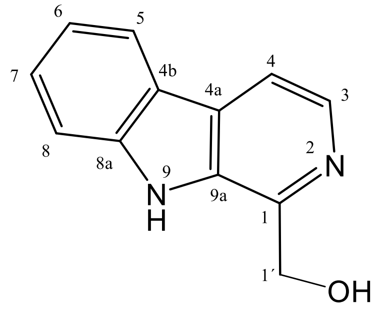

^{1}H NMR (400.13 MHz, DMSO) δ 11.36 (s, 1H), 8.24 (d, *J* = 5.1 Hz, 1H), 8.21 (d, *J* = 8.0 Hz, 1H), 8.01 (d, *J* = 5.1 Hz, 1H), 7.66 (d, *J* = 8.3 Hz, 1H), 7.52 (dd, *J* = 8.3 Hz, *J* = 7.5 Hz, 1H), 7.22 (dd, *J* = *J* = 8.0 Hz, 7.5 Hz, 1H), 4.96 (s, 2H). ^{13}C NMR (100.62 MHz, DMSO) δ 144.93, 140.53, 136.85, 133.46, 127.92, 127.88, 121.51, 120.54, 119.11, 113.75, 112.24, 63.53.

Table 1. NMR data of compound HA-5.

Position	^{13}C (ppm)	^{1}H (ppm)	integral	multiplicity	J (Hz)	COSY	^{1}H-^{13}C HMBC
**C-1**	144.93	-	-	-	-	-	
**C-3**	136.85	8.24	1H	d	5.13	8.01	144.93, 127.92
**C-4**	113.75	8.01	1H	d	5.13	8.24	136.85, 133.46
**C-4a**	127.92	-	-	-	-	-	
**C-4b**	120.54	-	-	-	-	-	
**C-5**	121.51	8.21	1H	d	8.00	7.22	140.53, 127.88
**C-6**	119.11	7.22	1H	dd	8.0,7.5	8.21, 7.52	120.54, 112.24
**C-7**	127.88	7.52	1H	dd	8.3,7.5	7.66, 7.22	140.35, 121.51
**C-8**	112.24	7.66	1H	d	8.25	7.52	120.54, 119.11
**C-8a**	140.53	-	-	-	-	-	
**C-9b**	133.46	-	-	-	-	-	
**1-CH$_2$OH**	63.53	4.96	2H	s	-	-	144.93, 133.46
	**^{15}N (ppm)**						
**N-2**	302	-	-	-	-	-	8.24, 8.01, 4.96*
**N-9**	115	11.36	1H	s	-	-	140.53, 133.46, 127.92, 120.54

* from an ^{1}H-^{15}N HMBC experiment

The NMR spectra are shown in Figures and all NMR data are compiled in Table 1. The ^{1}H NMR spectrum shows six ^{1}H aromatic signals, namely two triplets at 7.22 and 7.52 ppm, integrating for 1H each, and then four doublets at 7.66, 8.01, 8.21 and 8.24 ppm (1H each), which are assigned to six inequivalent aromatic hydrogens. These protons are one-bond correlated to the carbon atoms at 119.11, 127.88, 112.24, 113.75, 121.51 and 136.85 ppm respectively, as per the HSQCED spectrum. According to the COSY and TOCSY experiments, two spins systems are detected, one constituted by the 8.01 and 8.24 ppm doublets, and another by the signals at 8.21, 7.22, 7.52 and 7.66 ppm. The value of the coupling constants (6-7 Hz) indicated *ortho* (vicinal) relationships. A ^{1}H singlet at 4.96 ppm (2H), HSQC-correlated to a ^{13}C signal at 63.53 ppm, is attributed to a methylene (CH_2) group linked to an oxygen atom. A sharp ^{1}H singlet at 11.36 ppm (1H) is consistent with a pyrrole-like hydrogen. The ^{13}C peaks at 120.54, 127.92, 133.46, 140.53 and 144.93 ppm are thought to be due to quaternary aromatic carbon atoms. The ^{1}H-^{13}C HMBC experiment showed long-range heteronuclear correlations between the ^{1}H singlet at 4.96 ppm with the quaternary carbons at 133.46 and 144.93 ppm, this supporting that the CH_2 group was part of a pseudo benzylic alcohol function. The pyrrolic NH at 11.36 ppm showed HMBC correlations to the quaternary carbons at 120.54, 127.92, 133.46 and 140.53 ppm. Other representative HMBC cross peaks were those between the ^{1}H doublet at 8.01 ppm and the quaternary carbon at 133.46 ppm, that between the ^{1}H triplet at 7.52 ppm and the ^{13}C peak at 140.53 ppm, and the one involving the ^{1}H doublet at 8.20 ppm and the ^{13}C peak at 140.53 ppm. A ^{1}H-^{15}N HMBC experiment showed long-range correlations between the protons at 4.96, 8.01 and 8.24 ppm and a ^{15}N signal at 302 ppm. This chemical shift value

would agree with a pyridine-type nitrogen atom. The same ^{1}H-^{15}N HMBC spectrum showed a one-bond correlation between the hydrogen at 11.36 ppm with a ^{15}N resonance at 114.8 ppm, corroborating the pyrrole-type NH functionality. The presence of a four-hydrogen spin system agrees with a fused benzene ring, while the other two-hydrogen spin system, alongside the observed ^{1}H-^{15}N HMBC correlations, is consistent with a fused ortho-hydroxymethyl pyridine.

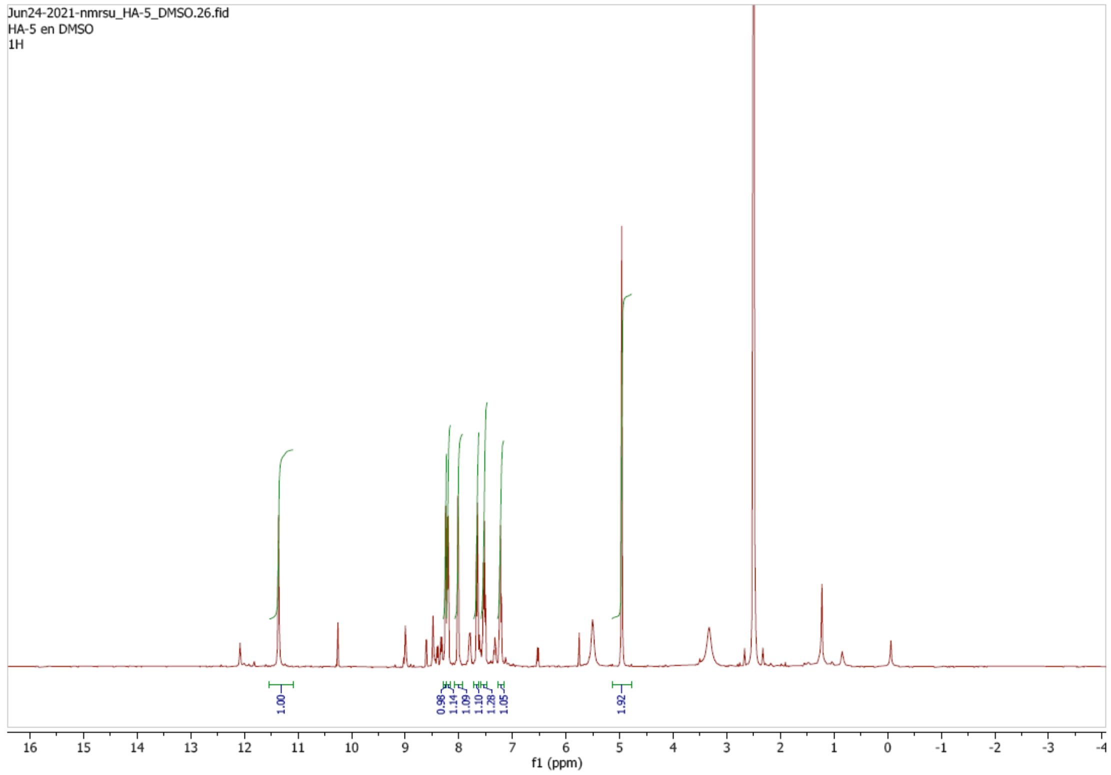

Figure 1a. ^{1}H NMR spectrum (400 MHz, DMSO-d_6) of compound HA-5

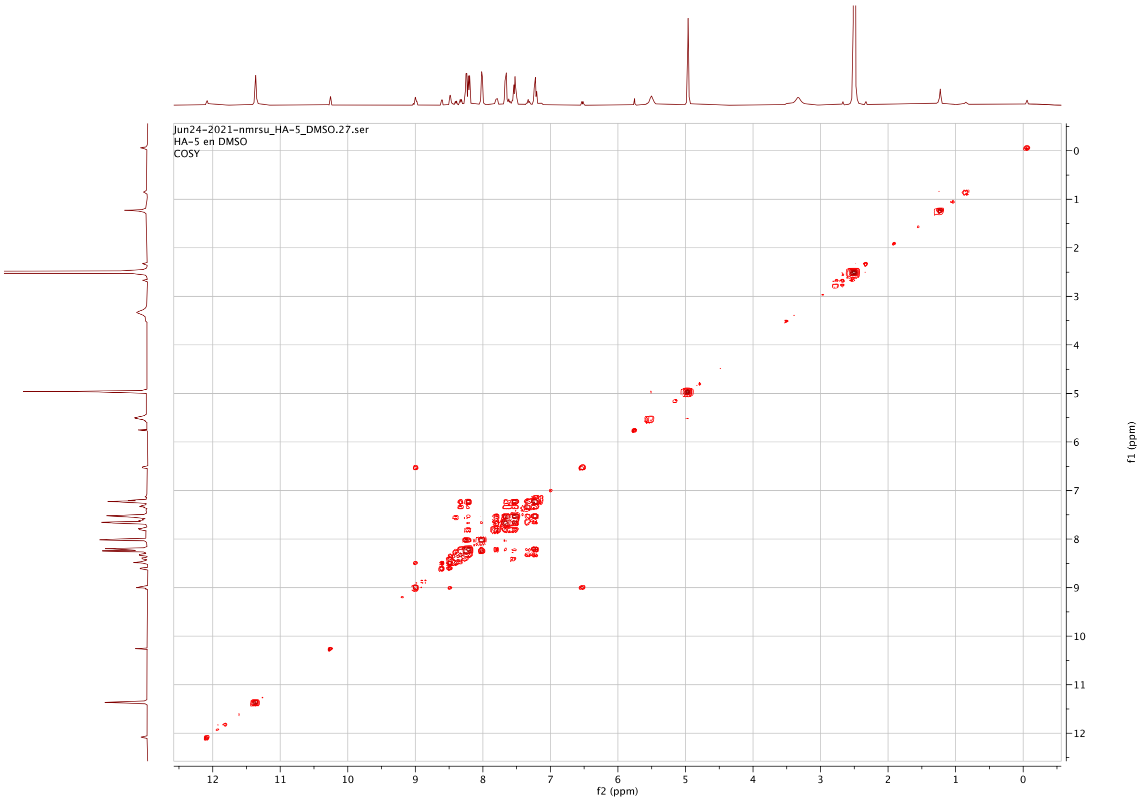

Figure 1b. COSY spectrum (400 MHz, DMSO-d$_6$) of compound HA-5

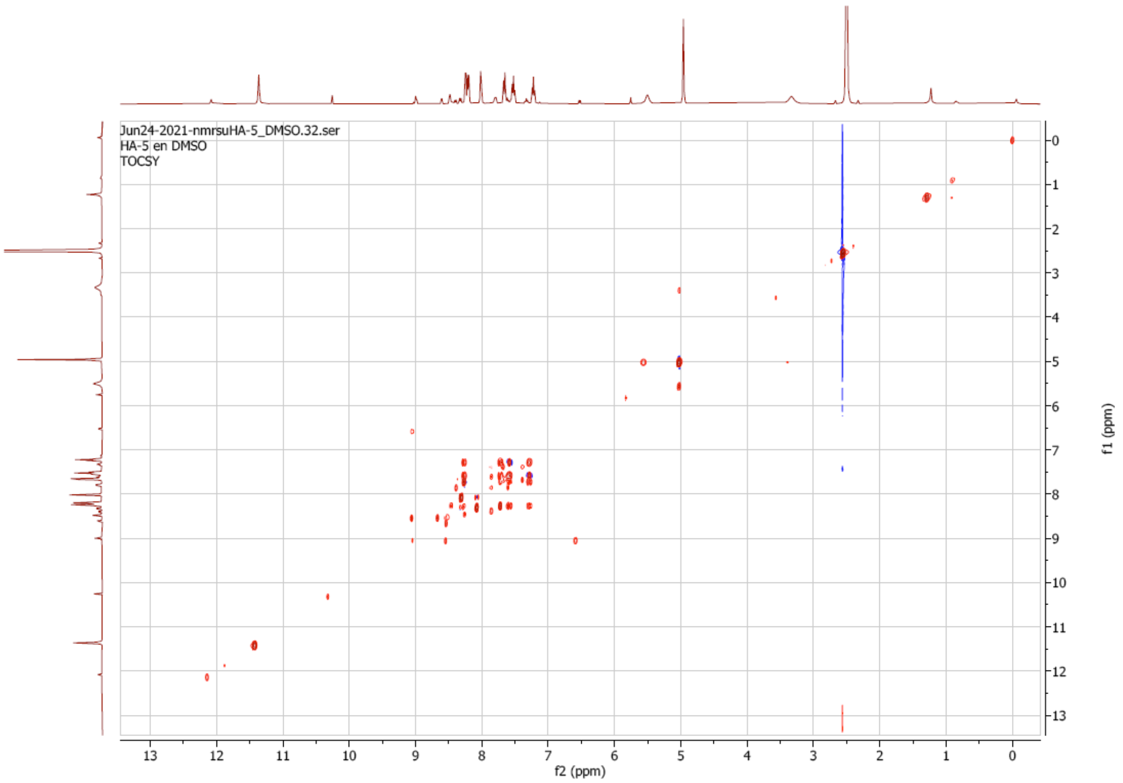

Figure 1c. TOCSY spectrum (400 MHz, DMSO-d$_6$) of compound HA-5

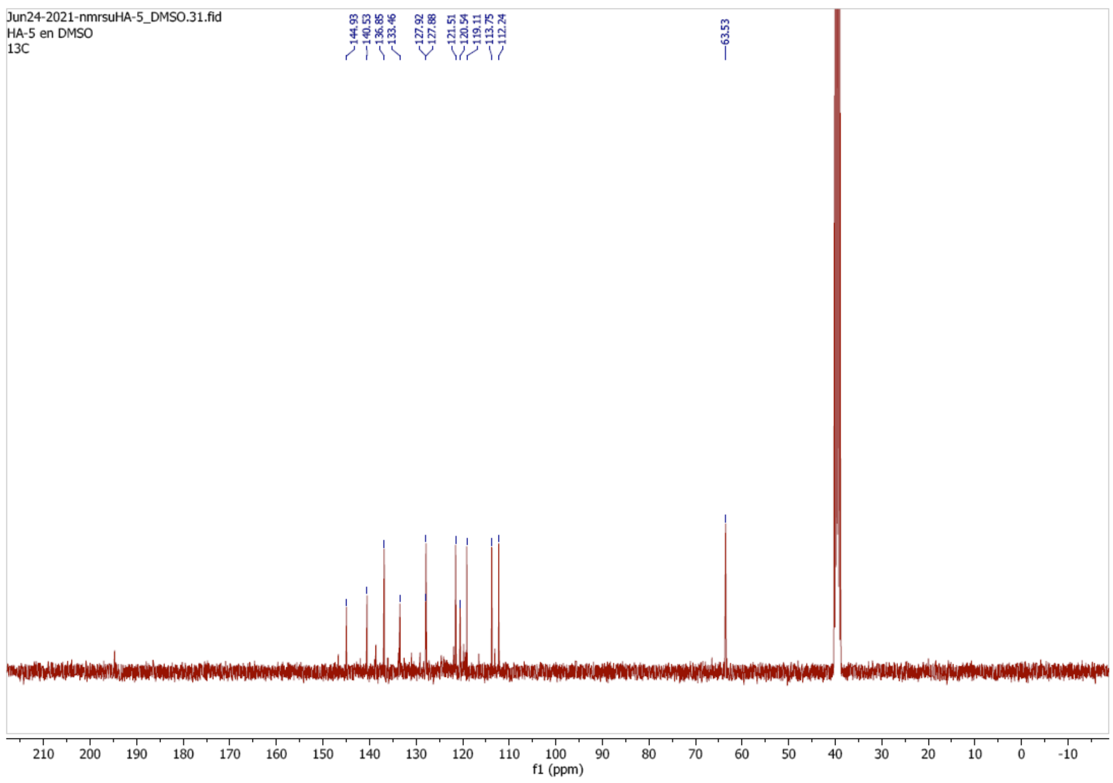

Figure 1d. ^{13}C NMR spectrum (100 MHz, DMSO-d_6) of compound HA-5

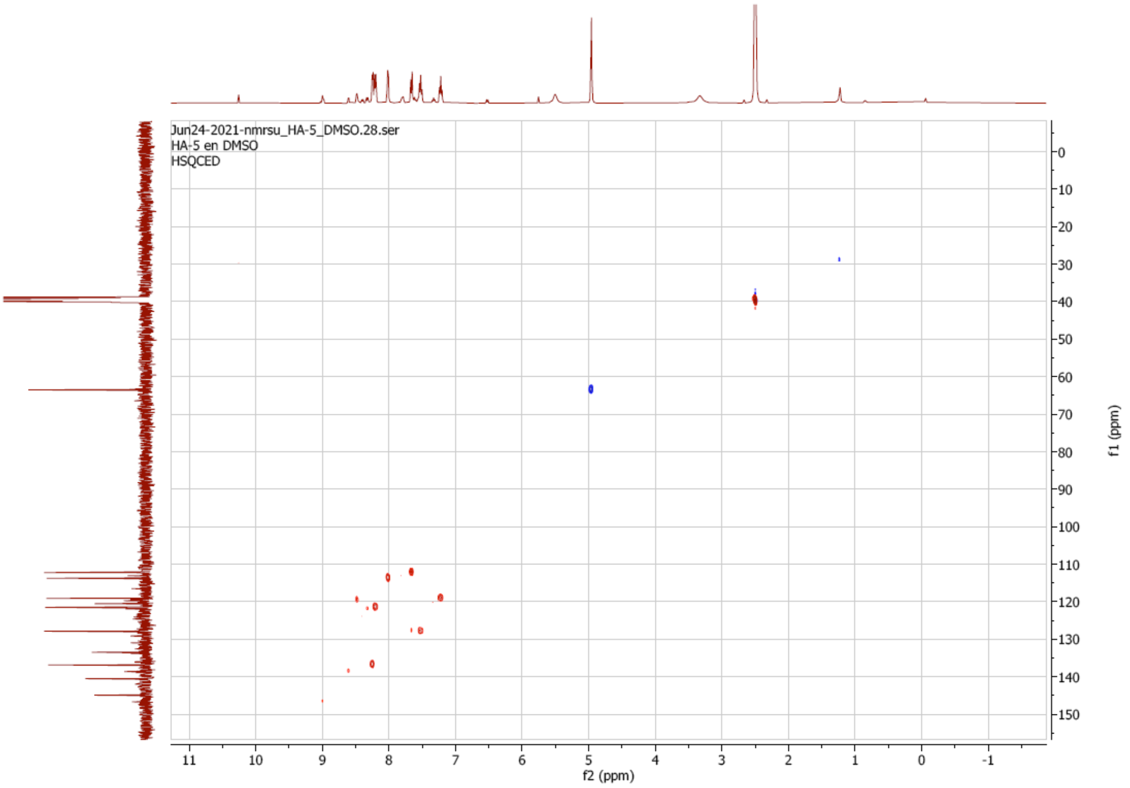

Figure 1e. ^{1}H-^{13}C HSQCED spectrum (400 MHz, DMSO-d_6) of compound HA-5.

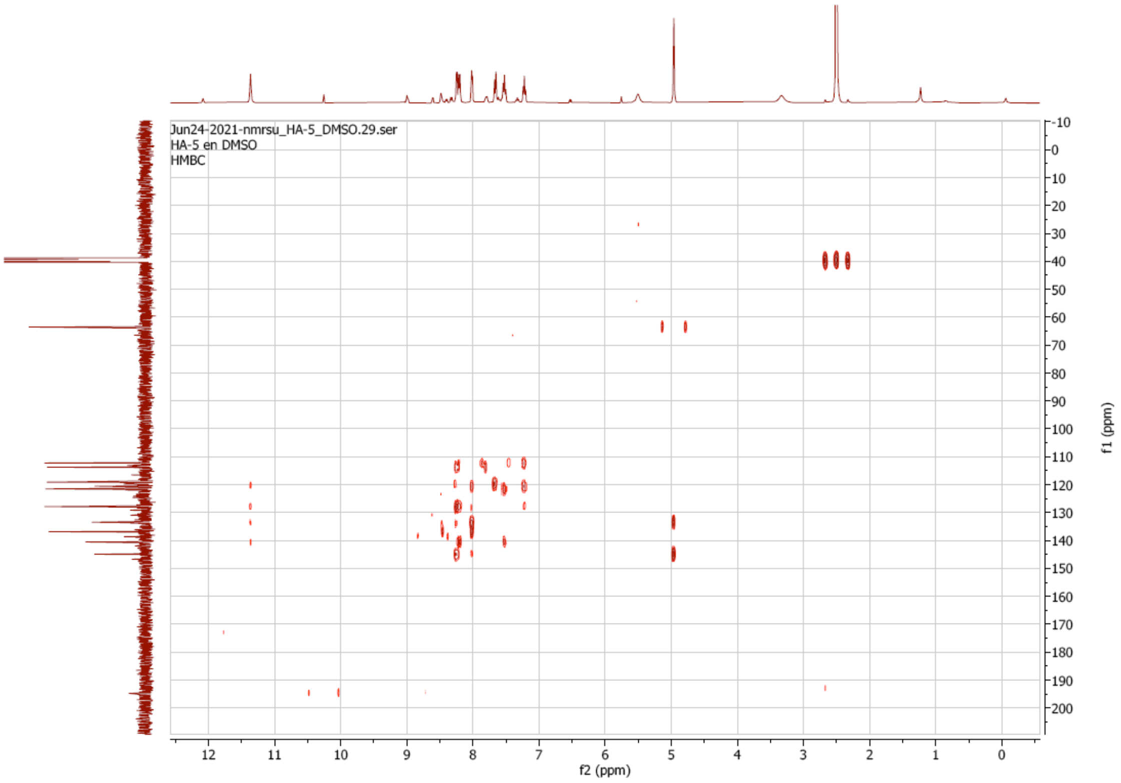

Figure 1f. ^{1}H-^{13}C HMBC spectrum (400 MHz, DMSO-d_6) of compound HA-5

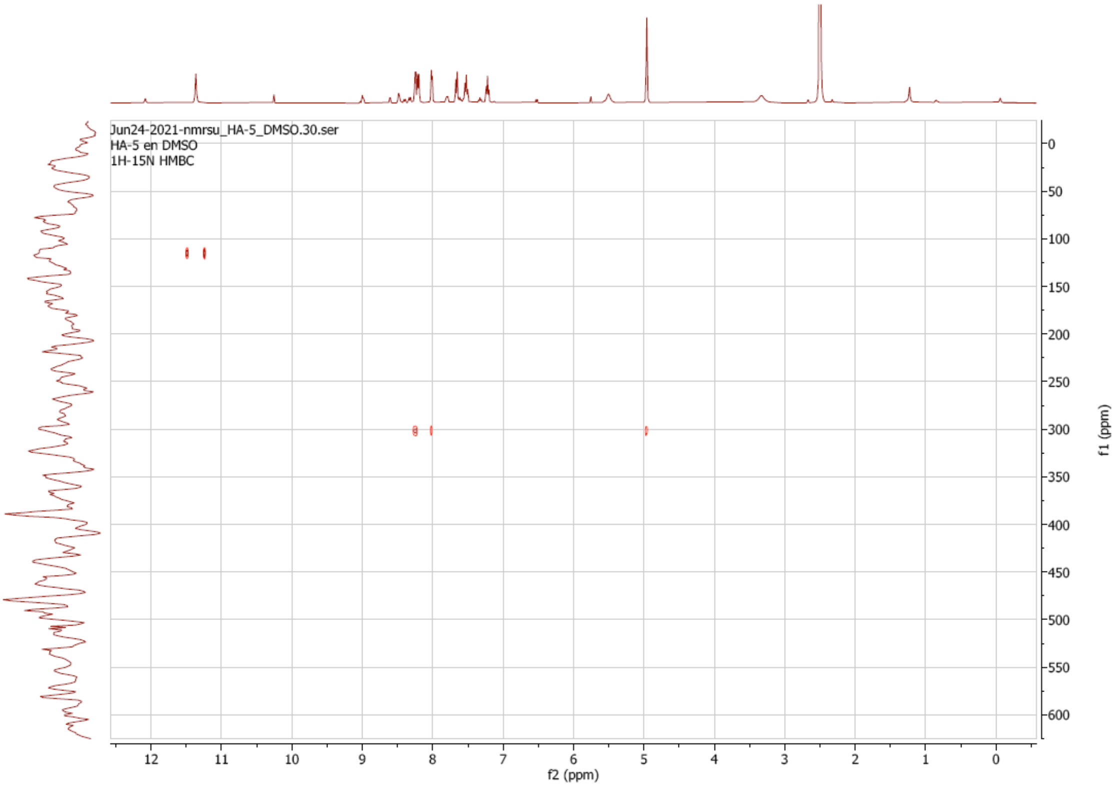

Figure 1g. ^{1}H-^{15}N HMBC spectrum (400 MHz, DMSO-d_6) of compound HA-5

2) (1-(1-hydoxyethyl)-β-carboline), ((1-(9*H*-pyrido[3,4-*b*]indol-1-yl)ethan-1-ol) (HET-βC; Comp. HA-6):

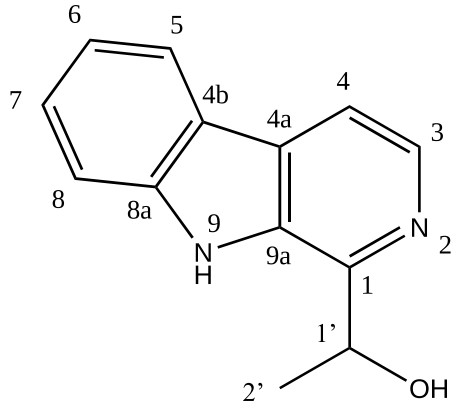

^{1}H NMR (400.13 MHz, DMSO) δ ^{1}H 11.23 (s, 1H), 8.23 (d, *J* = 4.6 Hz, 1H), 8.19 (d, *J* = 7.6 Hz, 1H), 7.99 (d, *J* = 4.6 Hz, 1H), 7.69 (d, *J* = 8.3 Hz, 1H), 7.51 (dd, *J* = 8.3 Hz, *J* = 7.7 Hz, 1H), 7.21 (dd, *J* = 7.6, *J* = 7.7 Hz, 1H), 5.68 (d, *J* = 3.6 Hz, 1H), 5.20 (m, 1H), 1.55 (d, *J* = 6.6 Hz, 3H). ^{13}C NMR (100.62 MHz, DMSO) δ ^{13}C 148.68, 140.49, 136.60, 132.26, 128.19, 127.77, 121.35, 120.44, 118.99, 113.47, 112.38, 69.32, 22.89.

Table 2. NMR data of compound HA-6

position	^{13}C (ppm)	^{1}H (ppm)	integral	multiplicity	J (Hz)	COSY	^{1}H-^{13}C HMBC
**C-1**	148.68	-	-	-	-	-	
**C-3**	136.60	8.23	1H	d	4.55	7.99	148.68, 129.19, 113.47
**C-4**	113.47	7.99	1H	d	4.55	8.23	136.60, 132.26, 120.44
**C-4a**	128.19	-	-	-	-	-	
**C-4b**	120.44	-	-	-	-	-	
**C-5**	121.35	8.19	1H	d	7.59	7.20	140.49, 127.77
**C-6**	118.99	7.21	1H	dd	7.6, 7.7	8.19, 7.51	127.77, 120.44, 112.38
**C-7**	127.77	7.51	1H	dd	8.3,7.7	7.69, 7.21	140.49, 121.35
**C-8**	112.38	7.69	1H	d	8.25	7.51	127.77, 120.44, 118.99
**C-8a**	140.49	-	-	-	-	-	
**C-9b**	132.26	-	-	-	-	-	
**1-<u>C</u>H(OH)Me**	69.32	5.20	1H	m	-	5.68, 1.55	148.68, 132.26, 22.89
**1-CH(OH)<u>Me</u>**	22.89	1.55	1H	d	6.58	5.20	148.68, 69.32
**1-CH(O<u>H</u>)Me**	-	5.68	1H	d	3.61	5.20	148.68, 69.32, 22.89
	**^{15}N (ppm)**						
**N-2**	299.4	-	-	-	-	-	8.23, 7.99*
**N-9**	115	11.23	1H	s	-	-	140.49, 132.26, 128.19, 120.44

* from an ^{1}H-^{15}N HMBC experiment

The ^{1}H and ^{13}C NMR spectra looked similar to those of HA-5, and the same conclusions could be reached by direct comparison. Only the aliphatic part looked slightly different, with a ^{1}H

multiplet (unresolved quadruplet, integrating for 1H) at 5.20 ppm and a doublet (3H) at 1.55 ppm. These two signals were one-bond correlated to the carbon atoms at 69.32 and 22.89 ppm, respectively. These ^{1}H and ^{13}C chemical shifts values were consistent with a 1-hydroxyethyl residue linked to an aromatic ring. Again, two aromatic spin systems, one made by the resonances at 8.19, 7.21, 7.51 and 7.69 ppm and another one constituted by the two doublets at 8.23 and 7.99 ppm, were observed. A singlet at 11.23 ppm (1H) was also seen. The ^{13}C spectrum showed five signals attributed to quaternary carbon atoms, like HA-5. A ^{1}H-^{15}N HMBC experiment showed long-range correlations between the hydrogens at 7.99 and 8.23 ppm with a pyridine-type nitrogen atom (^{15}N δ 299.3 ppm). A one-bond ^{1}H-^{15}N correlation between the proton at 11.23 ppm with a nitrogen at 115.2 ppm confirmed the pyrrolic nature of that NH residue.

HA-6

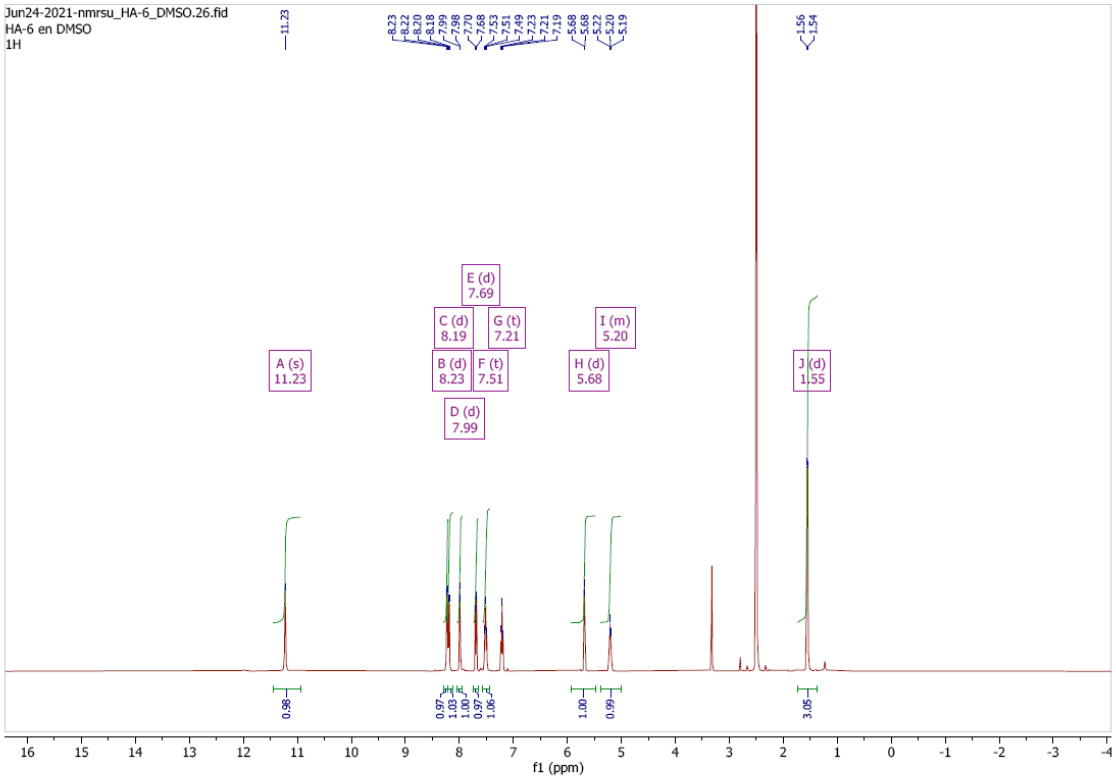

Figure 2a. ^{1}H NMR spectrum (400 MHz, DMSO-d_6) of compound HA-6

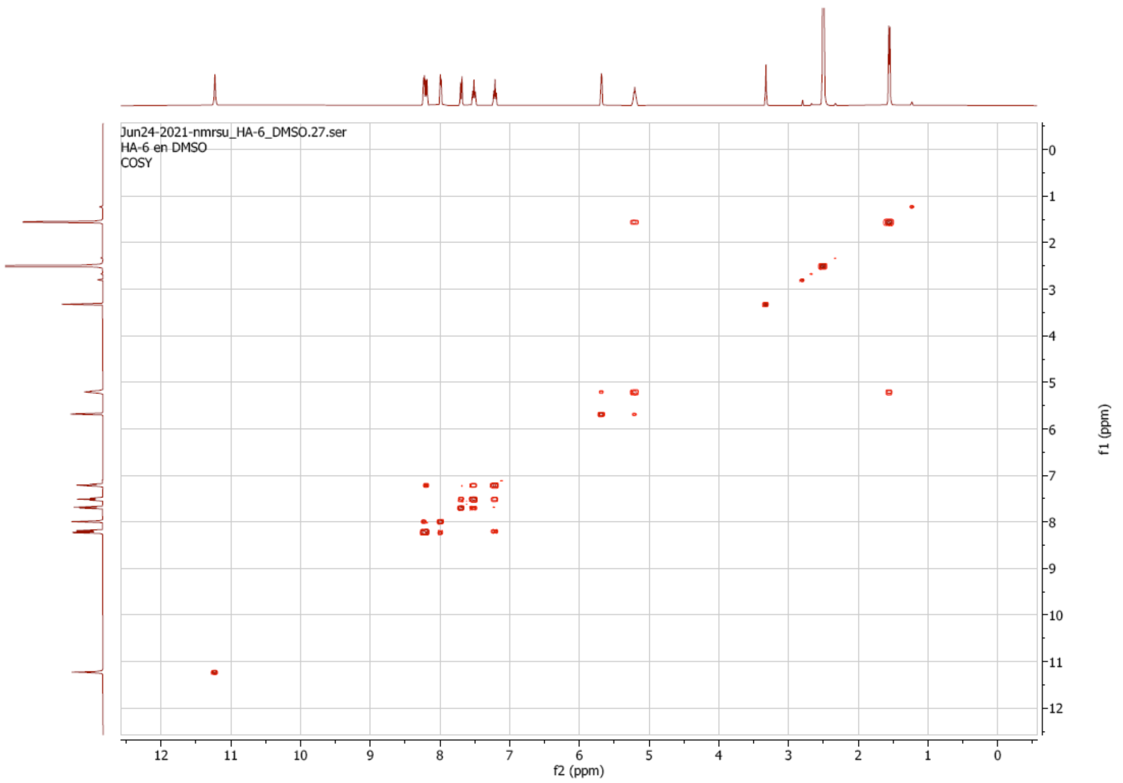

Figure 2b. COSY spectrum (400 MHz, DMSO-d$_6$) of compound HA-6

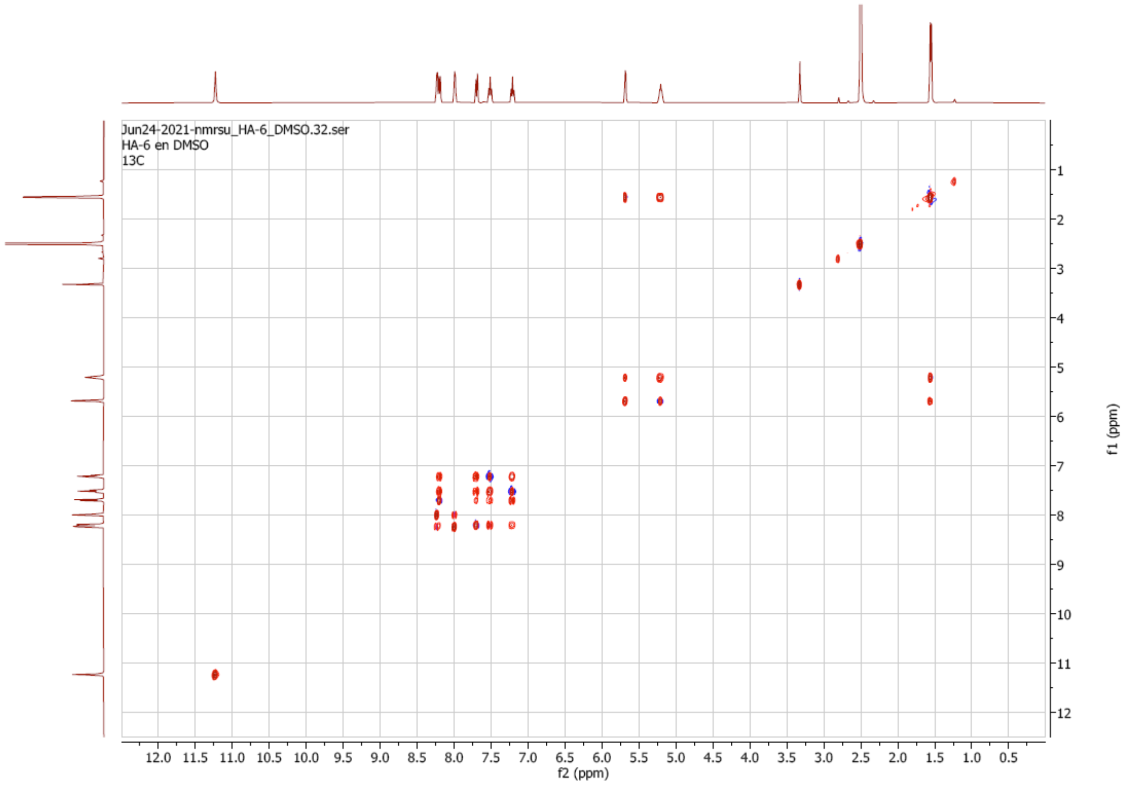

Figure 2c. TOCSY spectrum (400 MHz, DMSO-d$_6$) of compound HA-6.

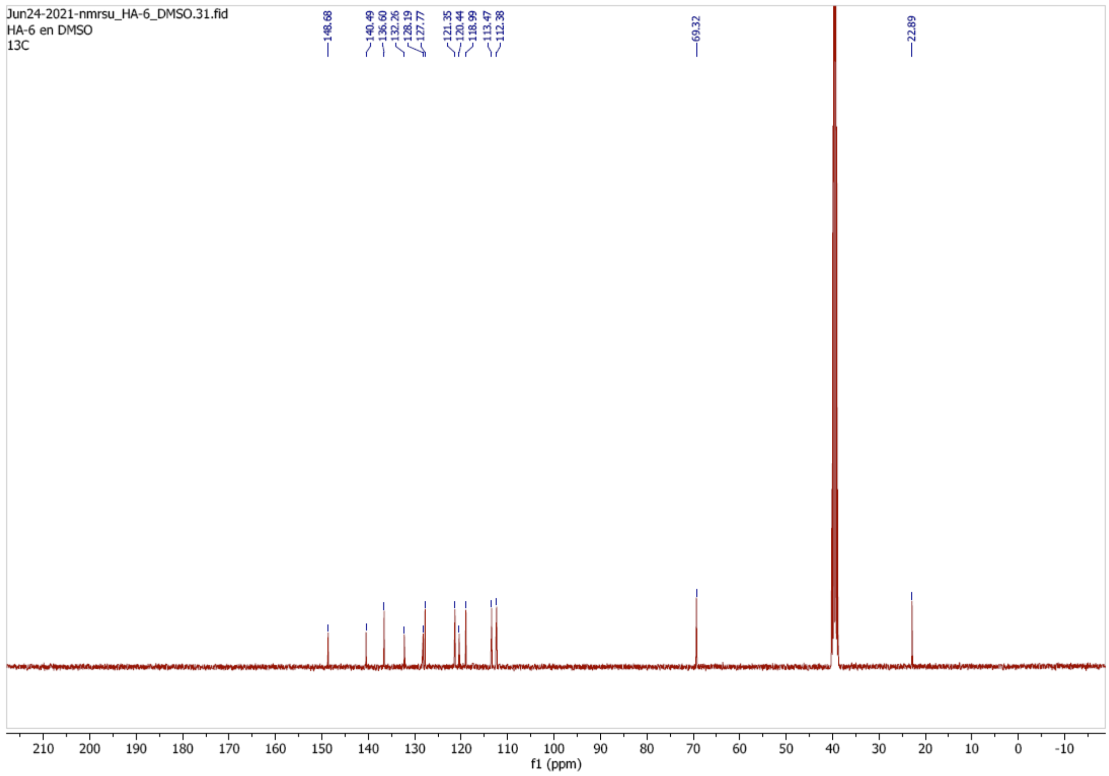

Figure 2d. ^{13}C NMR spectrum (100 MHz, DMSO-d_6) of compound HA-6

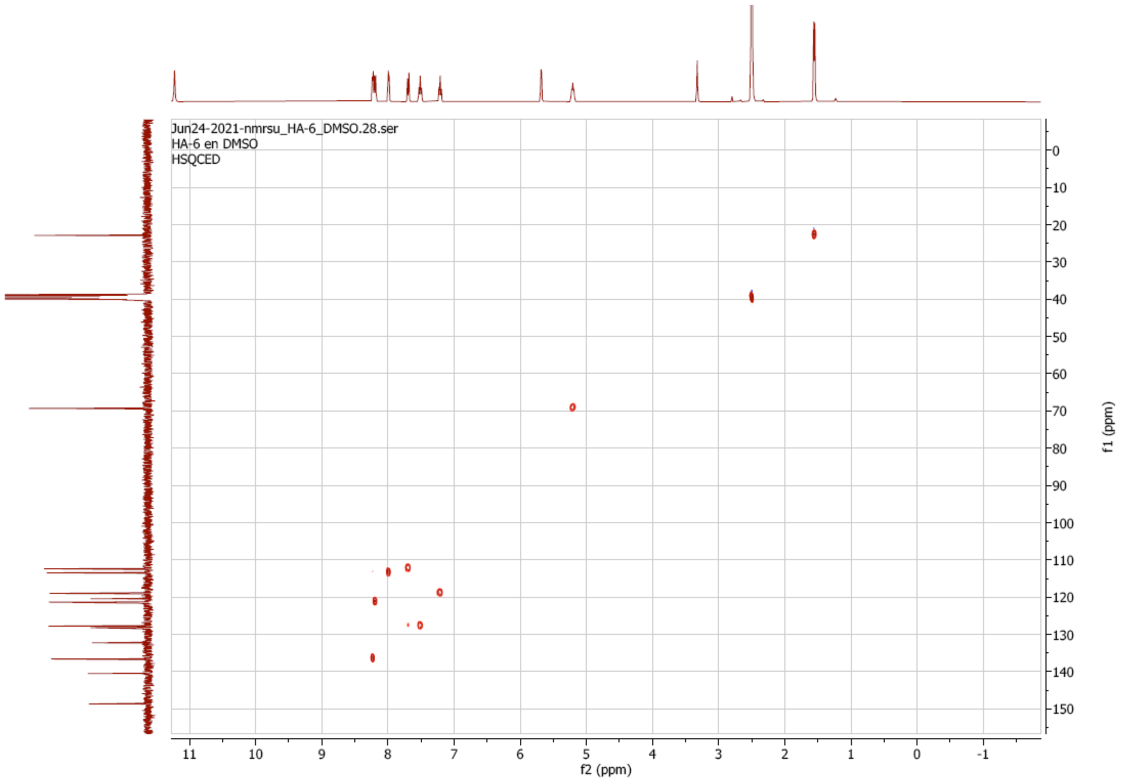

Figure 2e. ^1H-^{13}C HSQCED spectrum (400 MHz, DMSO-d_6) of compound HA-6.

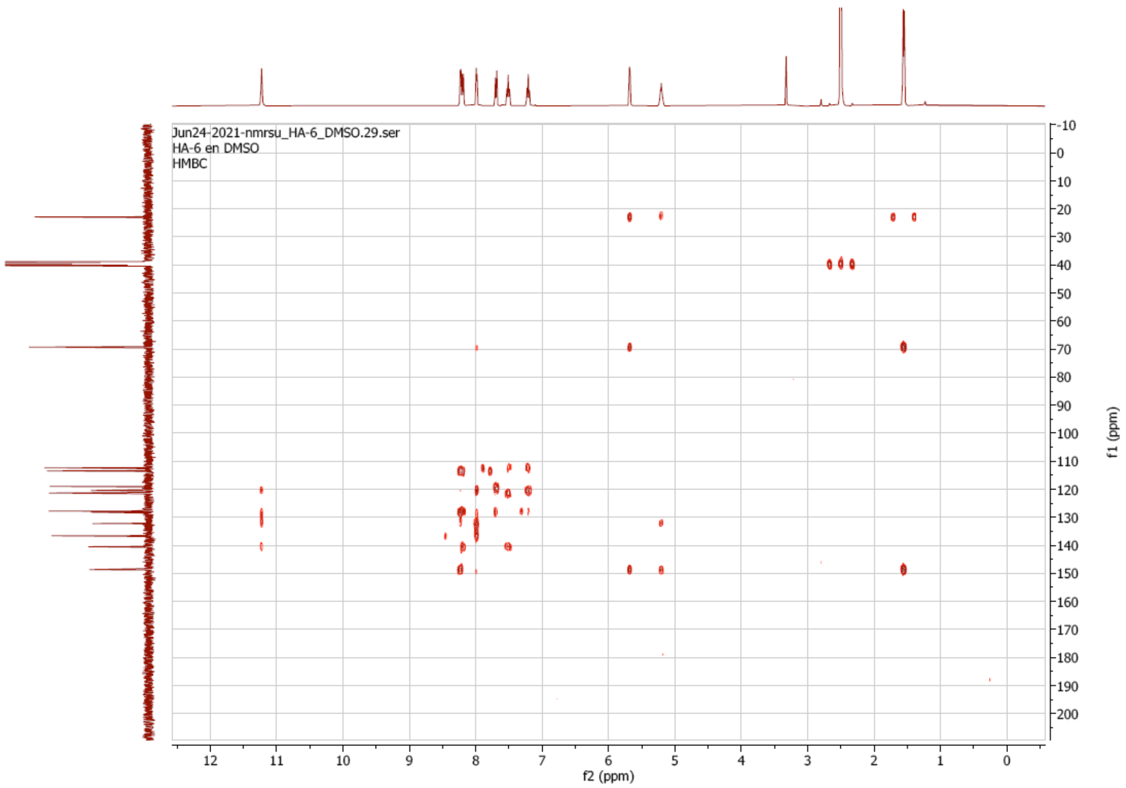

Figure 2f. ^{1}H-^{13}C HMBC spectrum (400 MHz, DMSO-d_6) of compound HA-6

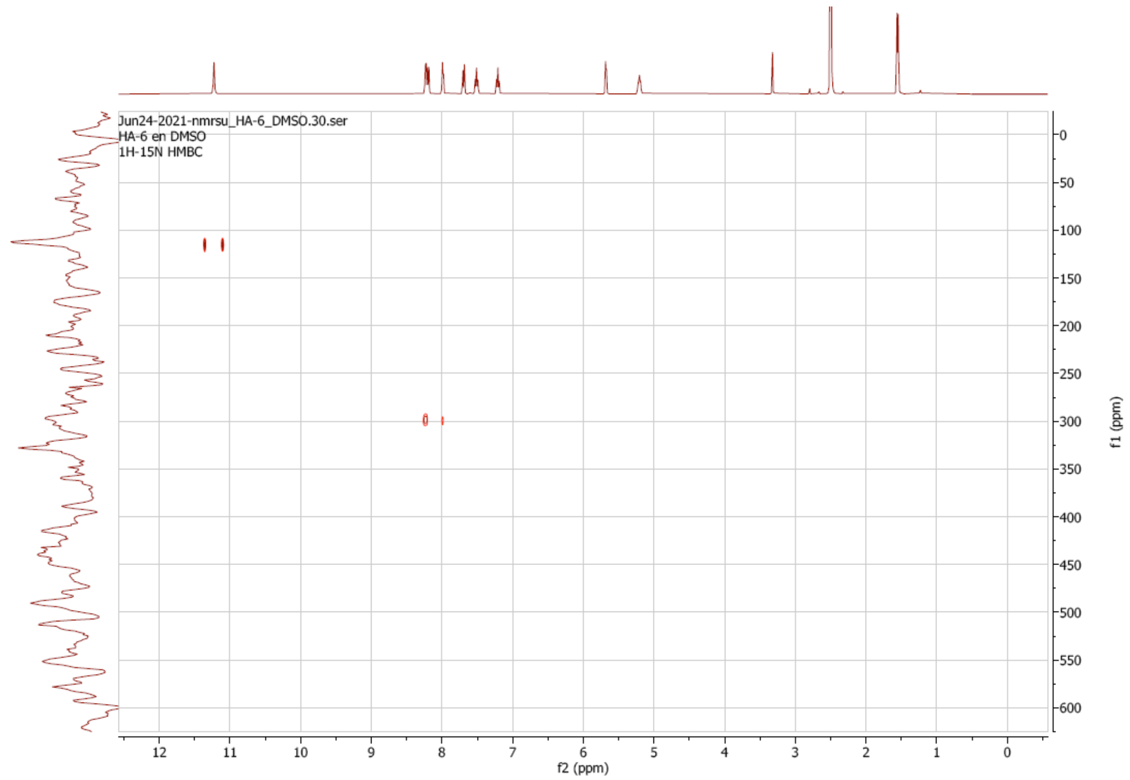

Figure 2g. ^{1}H-^{15}N HMBC spectrum (400 MHz, DMSO-d_6) of compound HA-6

3) (1-hydroxymethyl-β-carboline-3-carboxylic acid); ((1-(hydroxymethyl)-9*H*-pyrido[3,4-*b*]indole-3-carboxylic acid); (HME-βC-3-COOH); (Comp. AD-01):

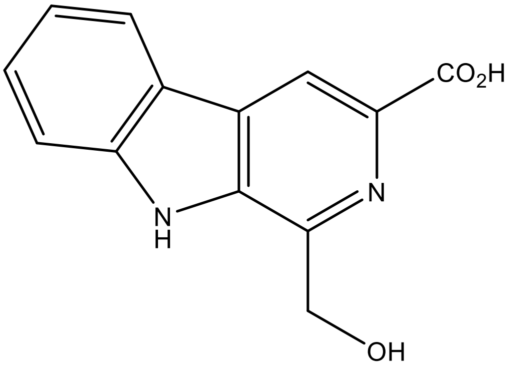

^{1}H NMR (400.13 MHz, DMSO) δ 11.89 (s, 1H), 8.84 (s, 1H), 8.37 (d, *J* = 7.8 Hz, 1H), 7.71 (d, *J* = 8.4 Hz, 1H), 7.59 (dd, *J* = 8.2 Hz, *J* = 7.2 Hz, 1H), 7.30 (dd, *J* = 7.8 Hz, *J* = 7.2 Hz, 1H), 5.03 (s, 2H). ^{13}C NMR (100.62 MHz, DMSO) δ 167.15, 144.74, 141.53, 135.32, 133.38, 129.41, 128.98, 122.48, 121.37, 120.59, 116.77, 113.08, 63.39.

Table 3. NMR data of compound AD-01

position	^{13}C (ppm)	^{1}H (ppm)	integral	multiplicity	J (Hz)	COSY	^{1}H-^{13}C HMBC
**C-1**	144.74	-	-	-	-	-	-
**C-3**	135.32	-	-	-	-	-	-
**C-4**	116.77	8.84	1H	s	-	-	167.15, 135.32, 121.37
**C-4a**	129.41*	-	-	-	-	-	-
**C-4b**	121.37	-	-	-	-	-	-
**C-5**	122.48	8.37	1H	d	7.8	7.30	141.53, 129.49*, 128.98
**C-6**	120.59	7.30	1H	dd	7.8,7.2	8.37, 7.59	121.37, 113.08
**C-7**	128.98	7.59	1H	dd	8.2,7.2	7.71, 7.30	141.53, 122.48
**C-8**	113.08	7.71	1H	d	8.2	7.59	121.37, 120.59*
**C-8a**	141.53	-	-	-	-	-	-
**C-9b**	133.38	-	-	-	-	-	-
**1-CH_2OH**	63.39	4.96	2H	s	-	-	144.74, 135.32
**3-CO_2H**	167.15	-	-	-	-	-	-
	**^{15}N (ppm)**						
**N-2**	n.d.	-	-	-	-	-	-
**N-9**	n.d.	11.89	1H	s	-	-	-

* doubtful

The NMR spectra of compound AD-01 are shown in Figures 3a-3e and all NMR data are compiled in Table 3. The spectrum is very similar to that of compound HA-5, and most of the signal assignments are made by direct comparison. Four ^{1}H signals, those at 8.37 (doublet), 7.30 (triplet), 7.59 ppm (triplet) and 7.71 ppm (doublet), each integrating for 1H, are consistent with the four hydrogens of a fused benzene ring and are assigned to hydrogens H-5 to H-8, respectively, in the 9*H*-pyrido[3,4-*b*]indole parent scaffold. A singlet at 8.84 ppm (1H), HSQC-correlated to a ^{13}C signal at 116.67 ppm, is assigned to H-4. Another ^{1}H singlet, that at 4.96

ppm, HSQC-correlated to ^{13}C 63.39 ppm and integrating for 2H, is assigned to the methylene group of the hydroxymethyl group at position C-1. A ^{1}H singlet at 11.89 ppm (1H) is attributed to the pyrrol-like hydrogen at position N-9. The main differences to HA-5 arise on the substitution at position 3 of the 9*H*-pyrido[3,4-*b*]indole main system. A ^{13}C peak at 167.15 ppm is assigned to a carboxylic carbon atom. The HMBC experiment showed a long-range correlation of this carbon atom with H-4 (8.84 ppm), this supporting substitution at the C-3 position of the 9H-pyrido[3,4-b]indole. The rest of the ^{13}C assignments, in particular those of the other quaternary carbons, are made relying upon HMBC data and by direct comparison to HA-5.

AD-01

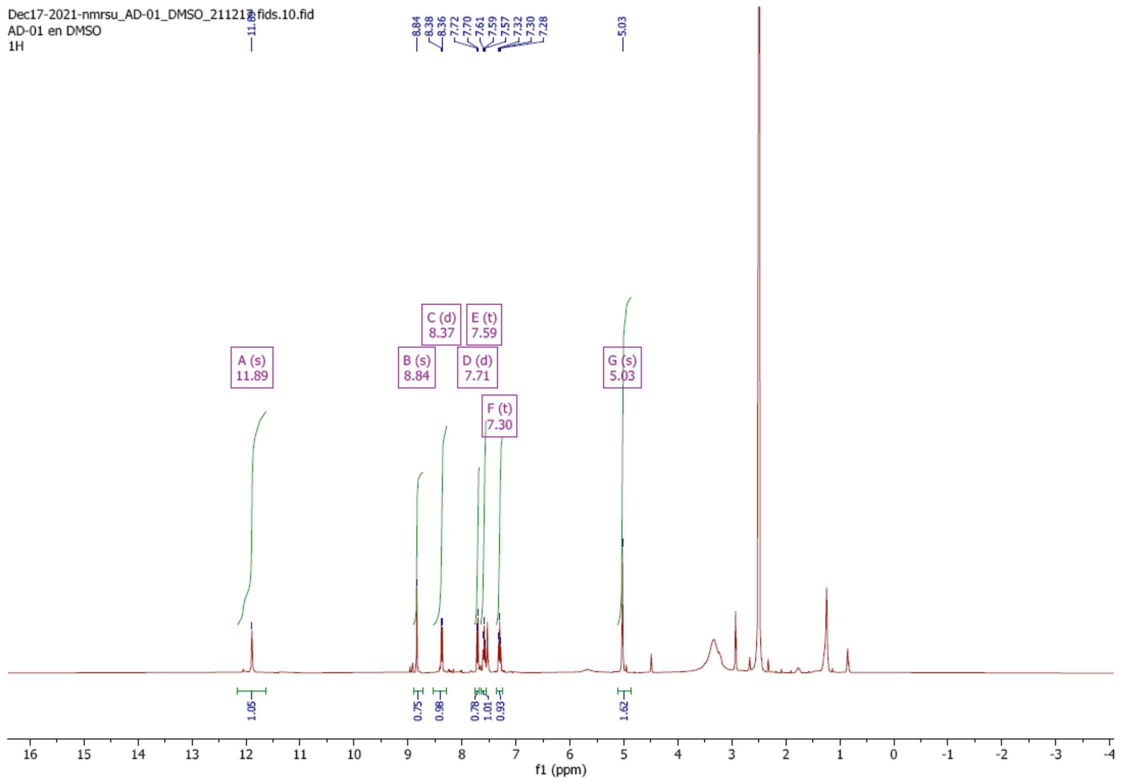

Figure 3a. ^{1}H NMR spectrum (400 MHz, DMSO-d_6) of compound AD-01.

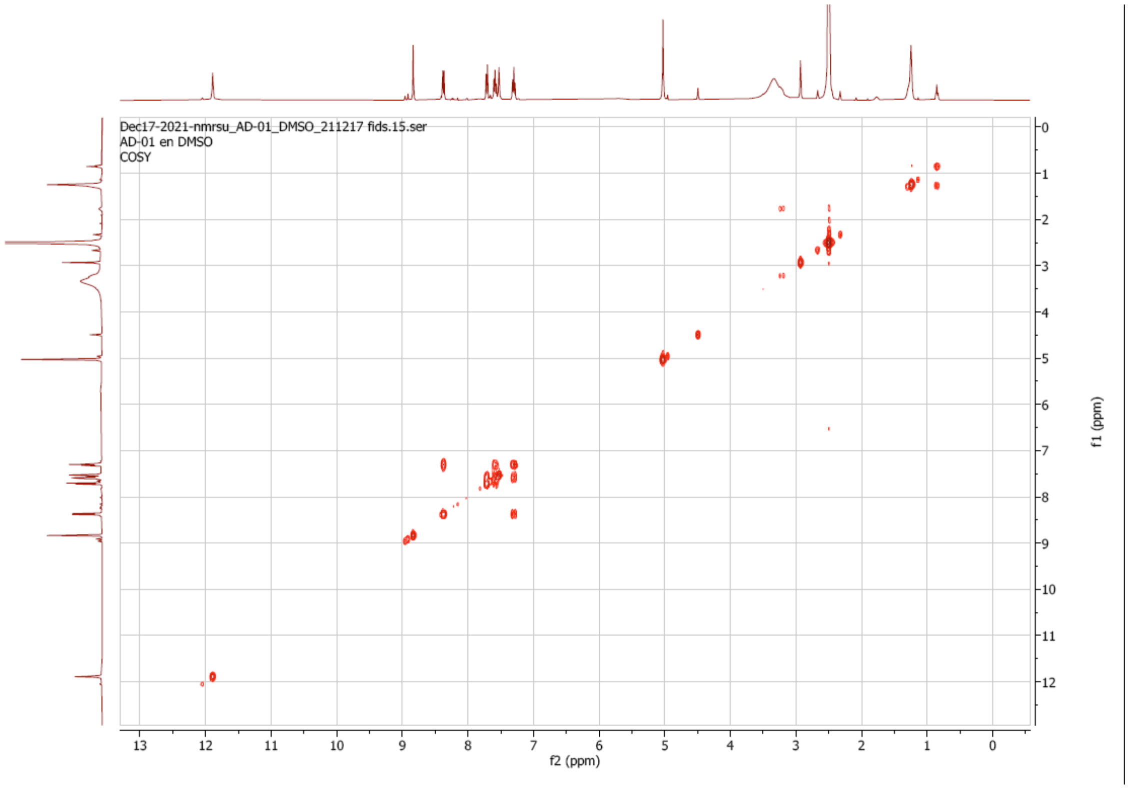

Figure 3b. COSY spectrum (400 MHz, DMSO-d_6) of compound HA-6

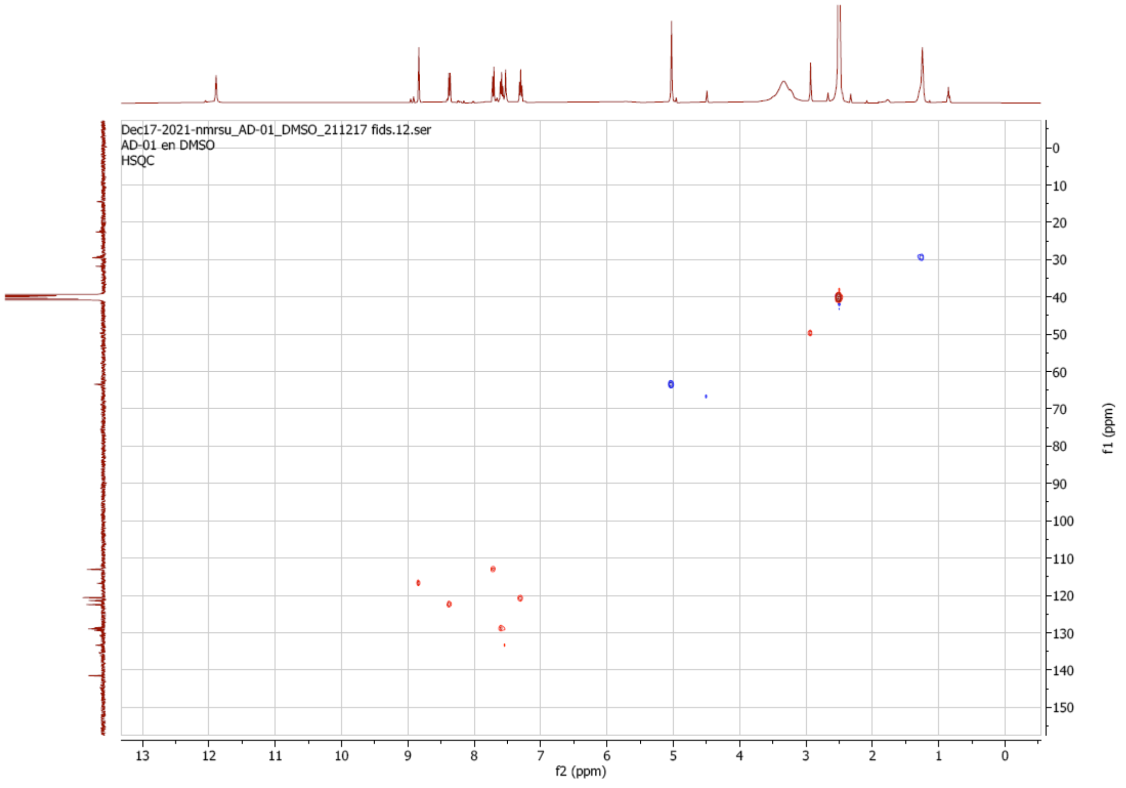

Figure 3c. ^{1}H-^{13}C HSQCED spectrum (400 MHz, DMSO-d_6) of compound AD-1

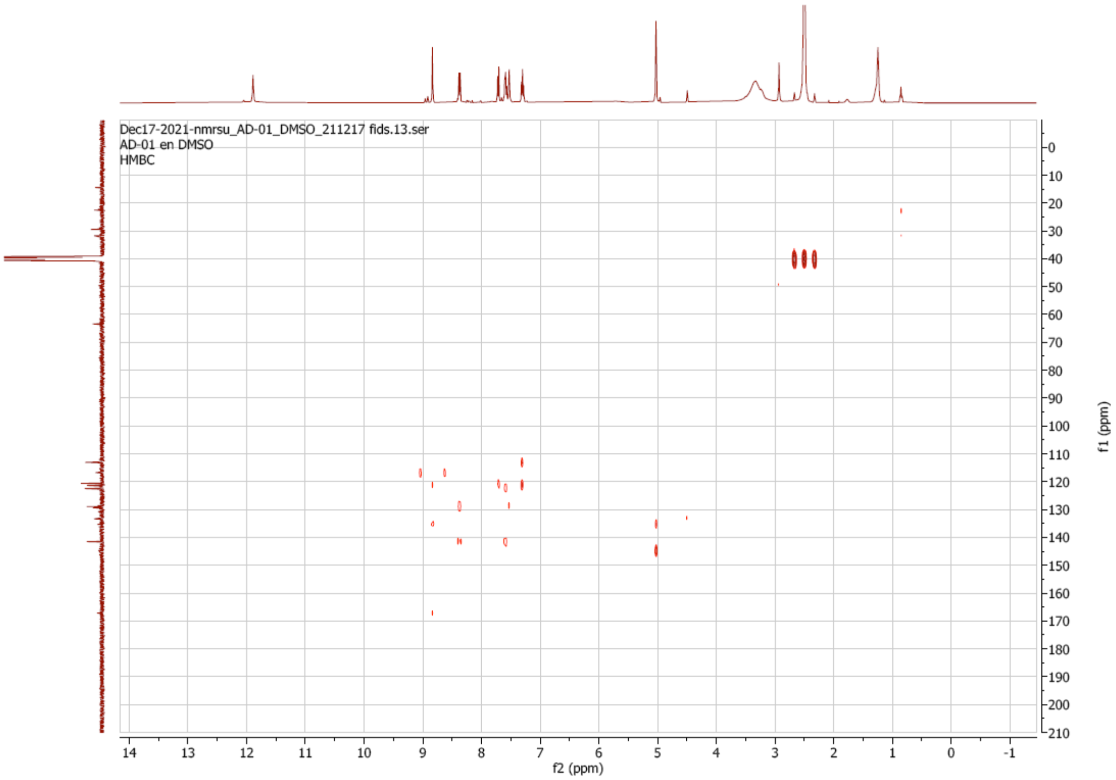

Figure 3d. ^{1}H-^{13}C HMBC spectrum (400 MHz, DMSO-d_6) of compound AD-01

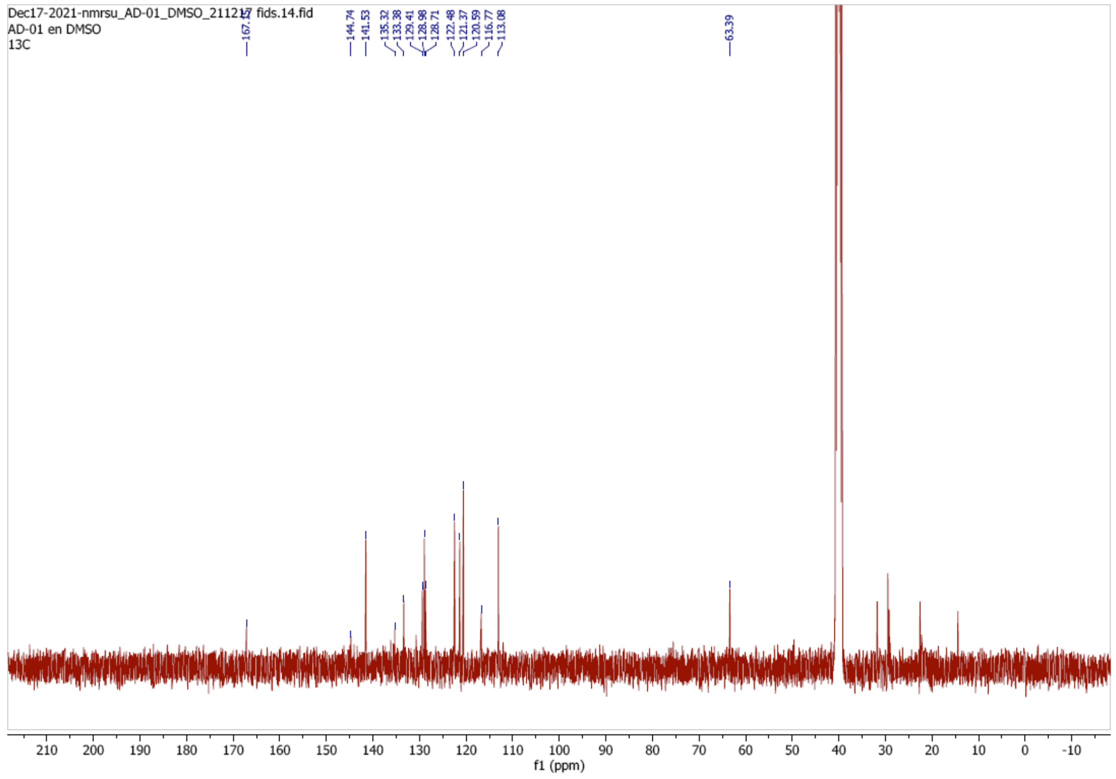

Figure 3e. ^{13}C NMR spectrum (100 MHz, DMSO-d_6) of compound AD-01

4) (1-(1-hydroxyethyl)-β-carboline-3-carboxylic acid); ((1-(hydroxyethyl)-9*H*-pyrido[3,4-*b*]indole-3-carboxylic acid); (HET-βC-3-COOH); (Comp. AD-02):

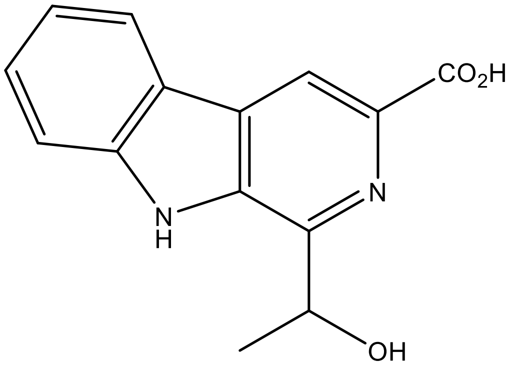

^{1}H NMR (400.13 MHz, DMSO) δ ^{1}H 11.77 (s, 1H), 8.81 (s, 1H), 8.36 (d, *J* = 7.9 Hz, 1H), 7.74 (d, *J* = 8.3 Hz, 1H), 7.58 (dd, *J* = 8.3 Hz, *J* = 7.6 Hz, 1H), 7.29 (dd, *J* = 7.9 Hz, *J* = 7.6 Hz, 1H), 5.28 (m, 1H), 1.57 (d, *J* = 6.6 Hz, 3H). ^{13}C NMR (100.62 MHz, DMSO) δ ^{13}C 166.78, 147.97, 141.06, ca. 135, 133.65, 128.60, 128.41, 121.84, 120.82, 119.99, 115.94, 112.71, 68.73, 23.09.

Table 4. NMR data of compound AD-02

**position**	**^{13}C (ppm)**	**^{1}H (ppm)**	**integral**	**multiplicity**	**J (Hz)**	**COSY**	**^{1}H-^{13}C HMBC**
**C-1**	147.97	-	-	-	-	-	-
**C-3**	135.82*	-	-	-	-	-	-
**C-4**	115.94	8.81	1H	s	-	-	166.78, 133.65, 120.82
**C-4a**	128.60	-	-	-	-	-	-
**C-4b**	120.82	-	-	-	-	-	-
**C-5**	121.84	8.36	1H	d	7.9	7.29	141.06, 128.41
**C-6**	119.99	7.29	1H	dd	7.9,7.6	8.36, 7.58	120.82, 112.71
**C-7**	128.41	7.58	1H	dd	8.3,7.6	7.74, 7.29	141.06, 121.84
**C-8**	112.71	7.74	1H	d	8.3	7.58	119.99
**C-8a**	141.06	-	-	-	-	-	-
**C-9b**	133.65	-	-	-	-	-	-
**1-CH(OH)Me**	68.73	5.28	1H	m	-	1.57	-
**1-CH(OH)Me**	23.09	1.57	3H	d	6.6	5.28	147.97, 68.73
**3-CO_2H**	166.78	-	-	-	-	-	-
	**^{15}N (ppm)**						
**N-2**	n.d.	-	-	-	-	-	-
**N-9**	n.d.	11.77	1H	s	-	-	-

* doubtful

The ^{1}H and ^{13}C NMR spectra looked very similar to those of AD-01 and HA-6, and the same conclusions on the structure could be dragged here. Only the aliphatic part looked slightly different, with a ^{1}H multiplet (unresolved quadruplet, integrating for 1H) at 5.28 ppm and a doublet (3H) at 1.57 ppm. These two signals were one-bond correlated to ^{13}C at 68.73 and 23.09 ppm, respectively, and were consistent with a 1-hydroxyethyl residue at the position C-1 of the 9H-pyrido[3,4-b]indole scaffold. The rest of the ^{1}H and ^{13}C resonances were essentially identical to those of AD-01 and were assigned by direct comparison. It was concluded that compound AD-2 was racemic (1-(hydroxyethyl)-9H-pyrido[3,4-b]indole-3-carboxylic acid.

AD-02

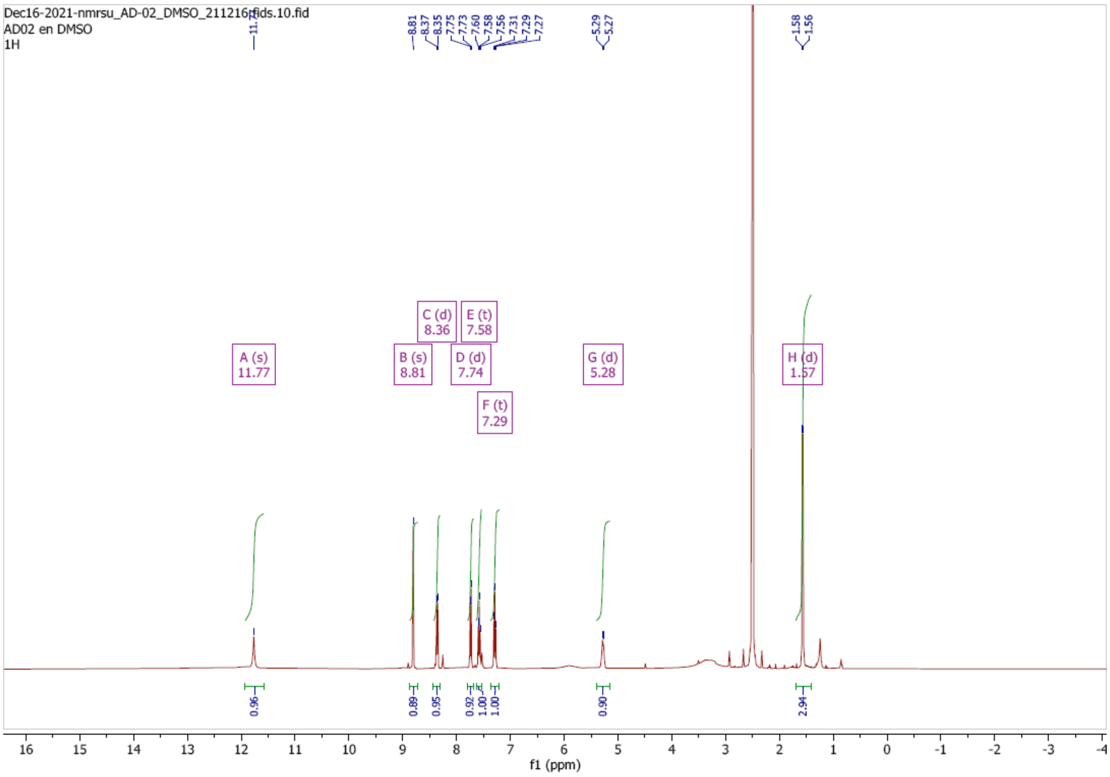

Figure 4a. ^{1}H NMR spectrum (400 MHz, DMSO-d_6) of compound AD-02

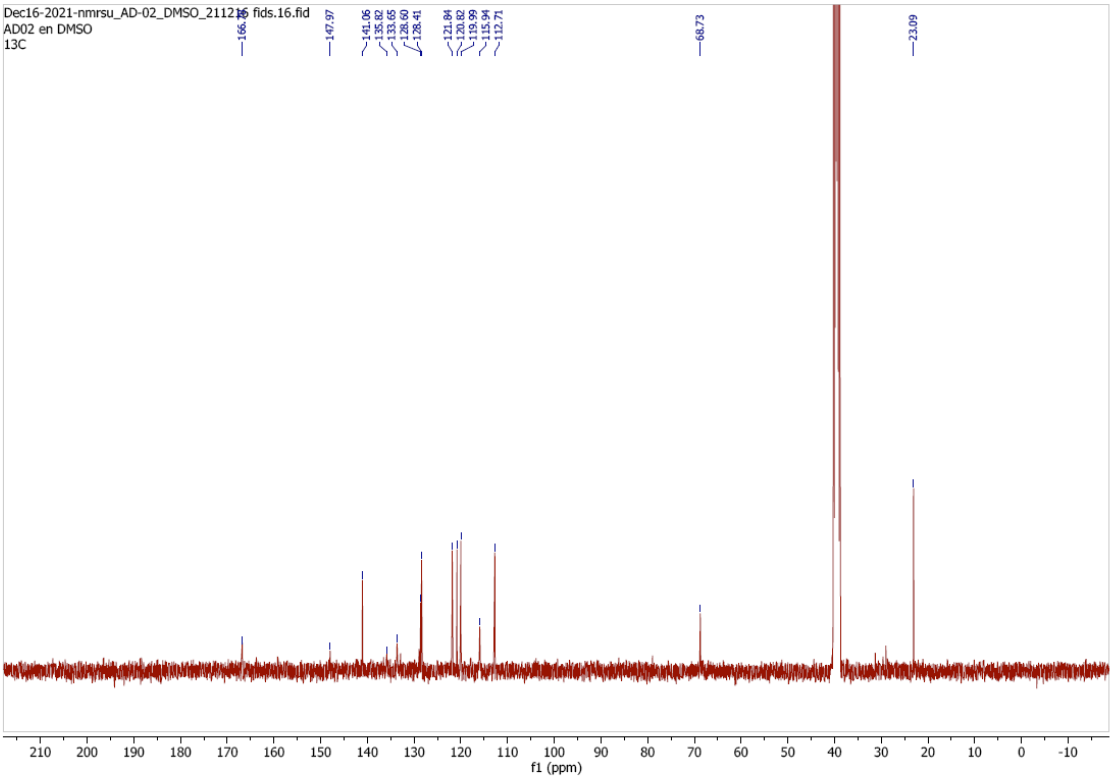

Figure 4b. ^{13}C NMR spectrum (400 MHz, DMSO-d_6) of compound AD-02

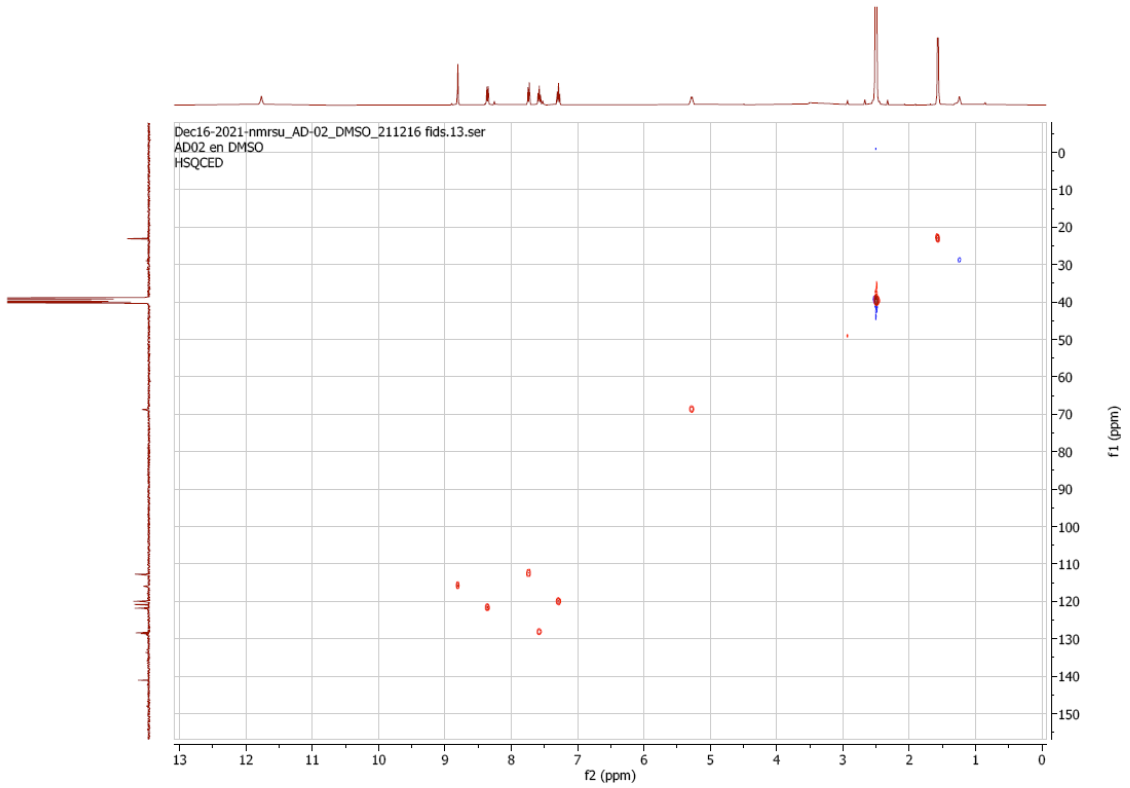

Figure 4c. ^{1}H-^{13}C HSQCED spectrum (400 MHz, DMSO-d_6) of compound AD-02

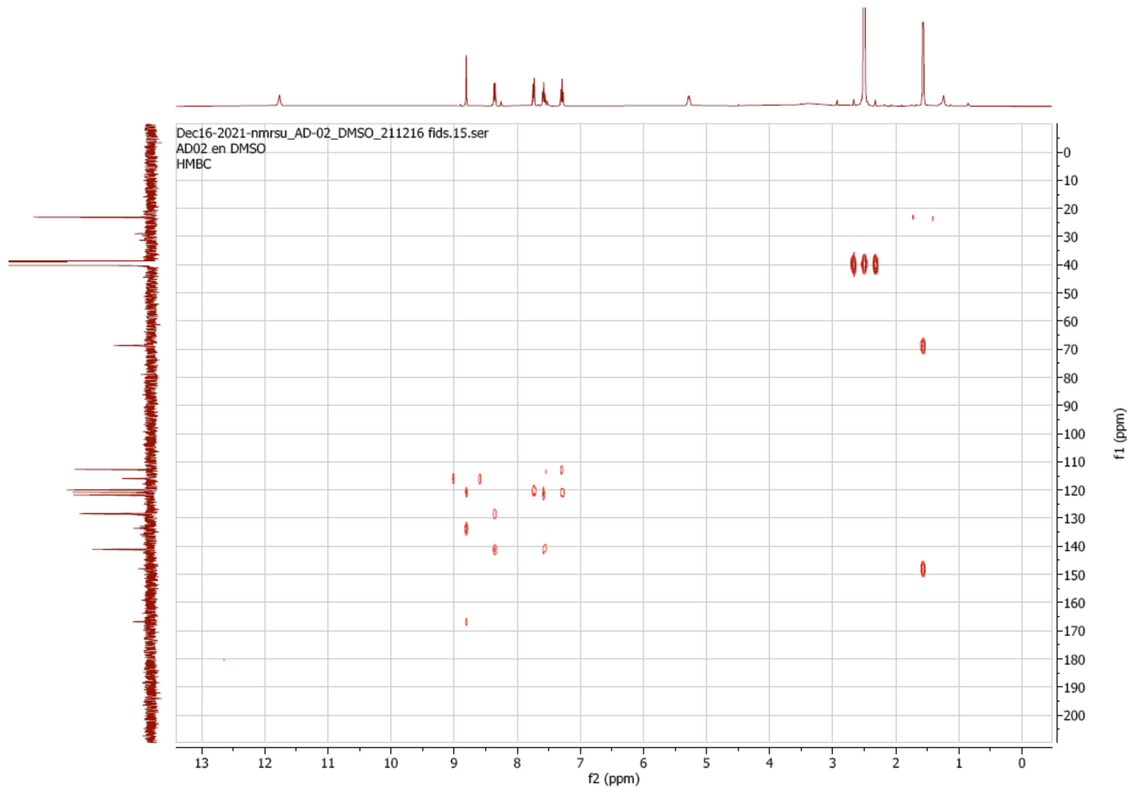

Figure 4d. ^{1}H-^{13}C HMBC spectrum (400 MHz, DMSO-d_6) of compound AD-02

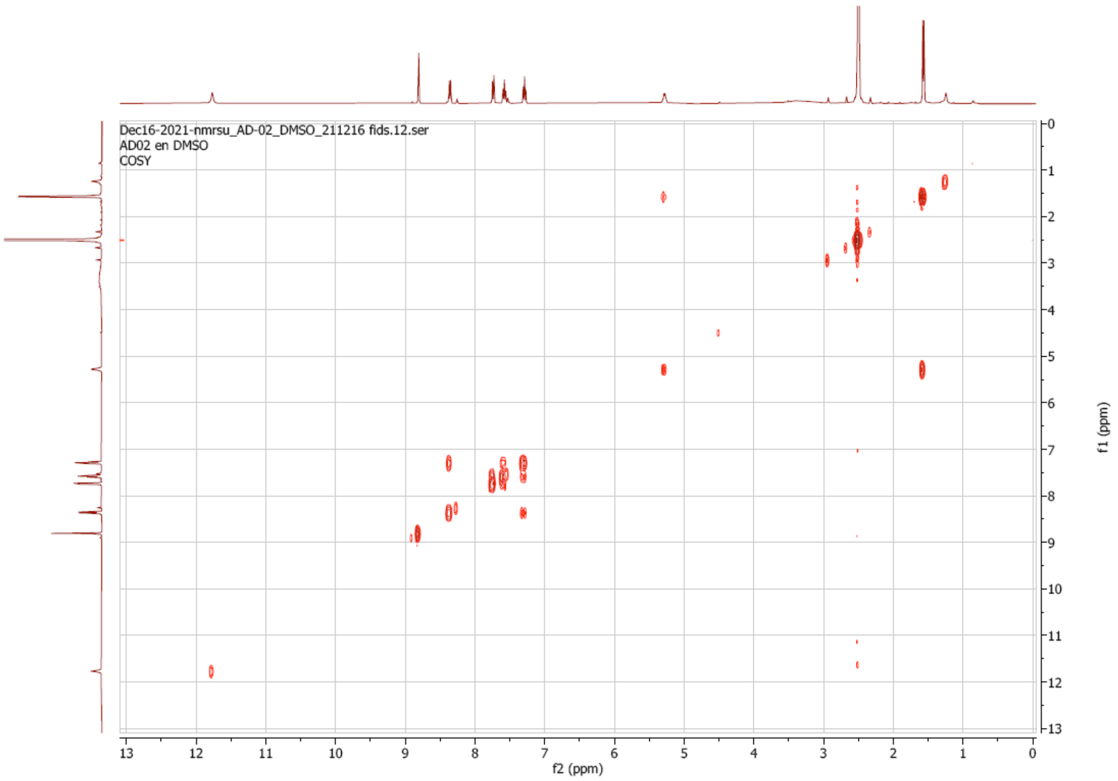

Figure 4e. COSY spectrum (400 MHz, DMSO-d_6) of compound AD-02

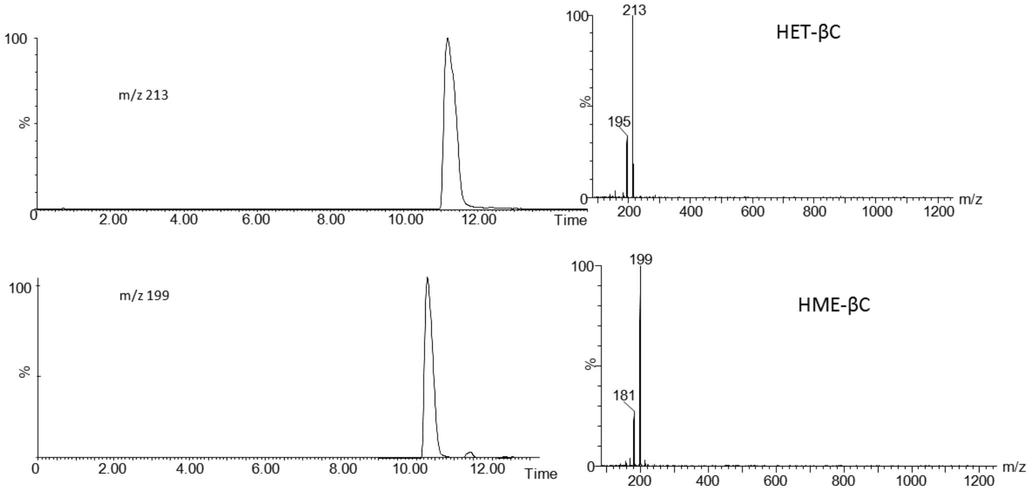

Figure 5. HPLC-MS (ESI+, 20 V) of HET-βC and HME-βC.

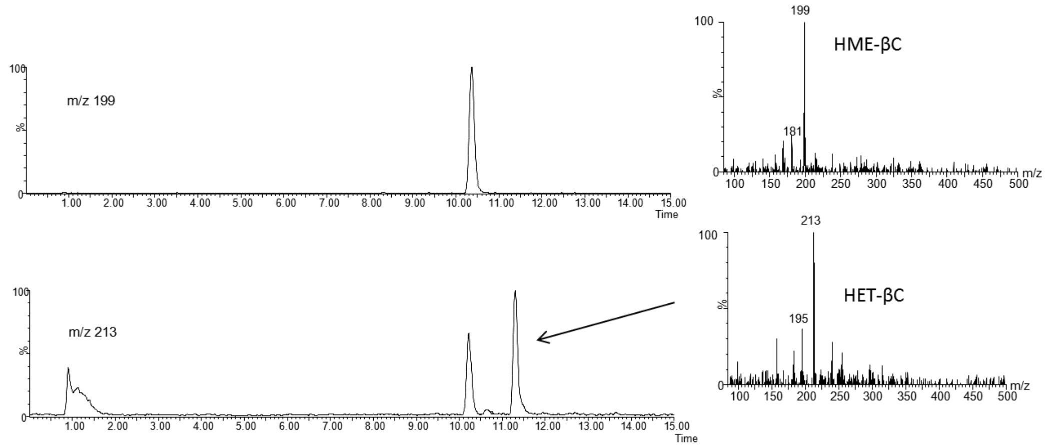

Figure 6. HPLC-MS (ESI+, 20 V) of HME-βC and HET-βC in model reactions of *L*-tryptophan (0.5 mg/mL) and fructose (4.6 mg/mL) (pH 2.85, 90 ºC, 20 h).

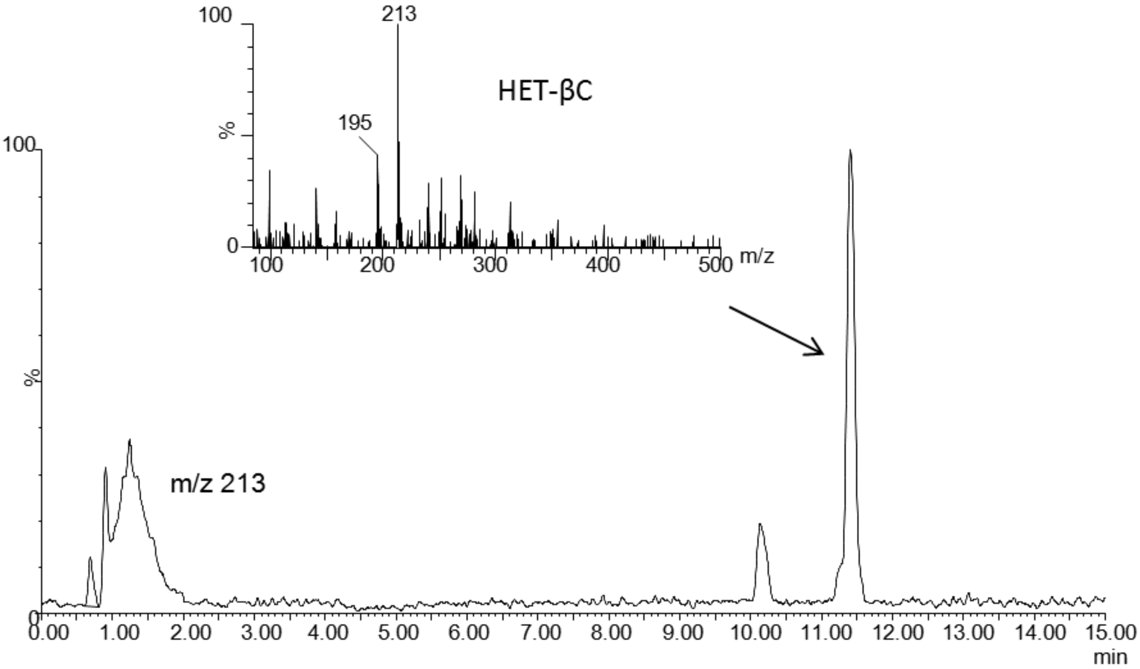

Figure 7. HPLC-MS (ESI+, 20 V) detection of HET-βC in the reaction of *L*-tryptophan (0.5 mg/mL) and 3-deoxyglucosone (0.1 mg/mL) (pH 3, 110 ºC, 2 h).

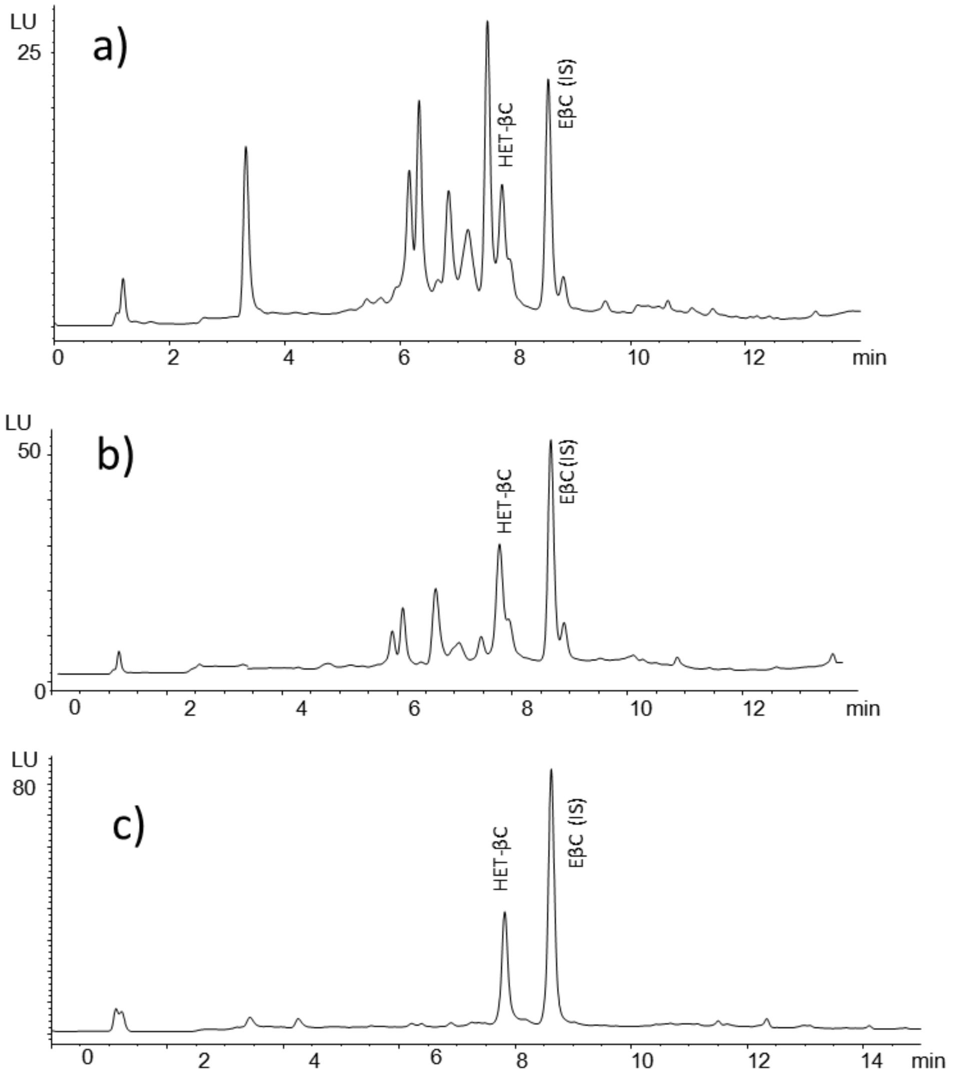

Figure 8. RP-HPLC chromatograms of food extracts. Crunchy dried tomatoes (a), crispy fried onion (b) and Manuka honey (c). Fluorescence detection (300 nm exc.; 433 nm emiss).

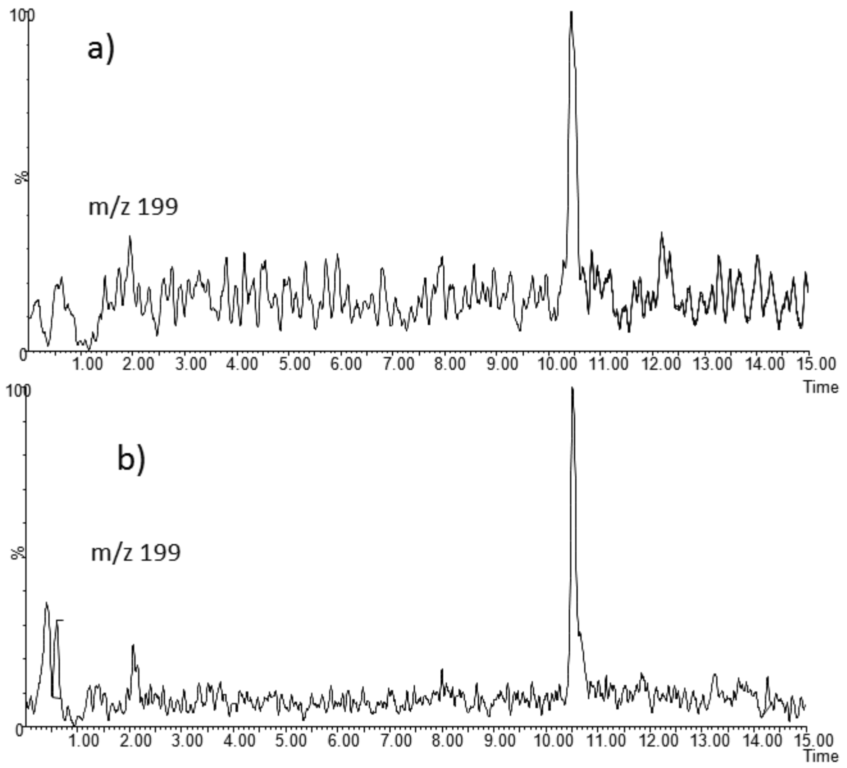

Figure 9. HPLC-MS (ESI+, 20 V) detection of HME-βC (*m/z* 199) in food extracts in crunchy dried tomatoes (a) and crispy fried onion (b).

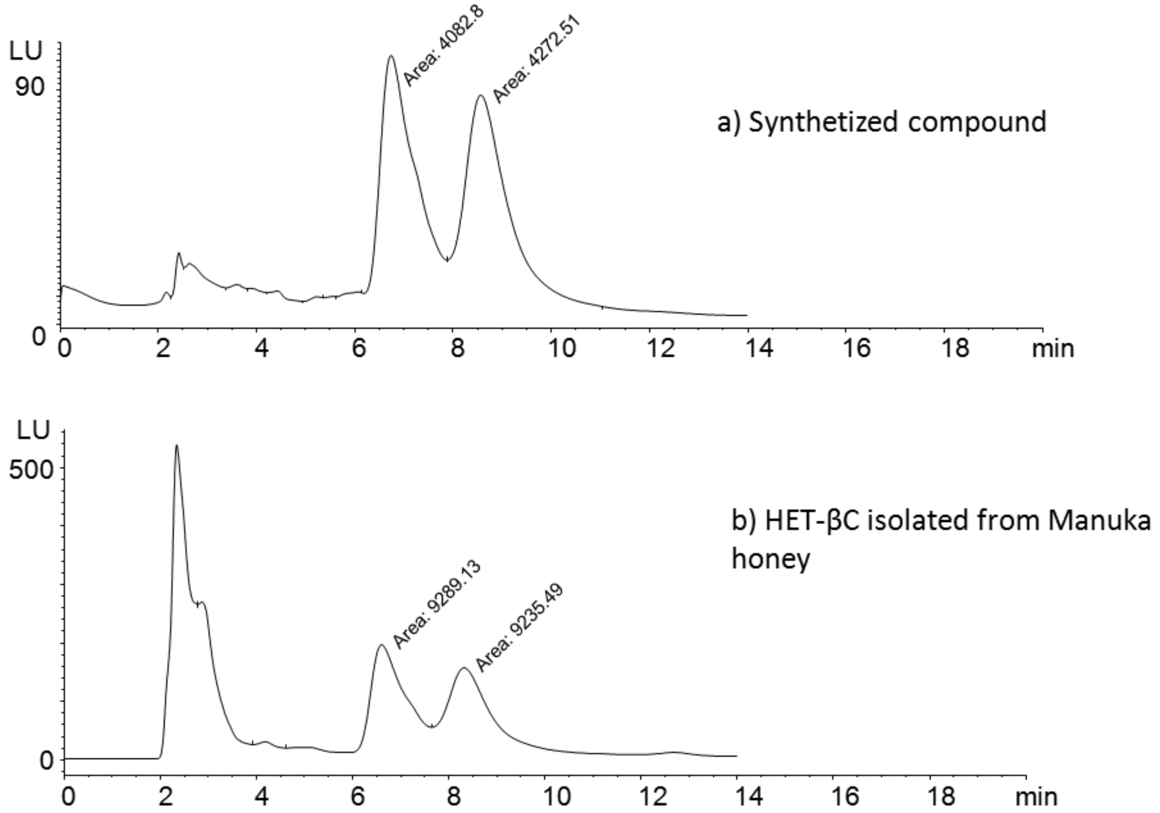

Figure 10. Chiral chromatography of synthesized HET-βC (a) and HET-βC isolated from Manuka honey (b) analysed into a column Chiralpak IA. Fluorescence detection (300 nm exc.; 433 nm emiss).
